# Triggered Drug Release From Liposomes: Exploiting the Outer and Inner Tumor Environment

**DOI:** 10.3389/fonc.2021.623760

**Published:** 2021-03-16

**Authors:** Marina Santiago Franco, Eliza Rocha Gomes, Marjorie Coimbra Roque, Mônica Cristina Oliveira

**Affiliations:** Department of Pharmaceutical Products, Faculty of Pharmacy, Universidade Federal de Minas Gerais, Belo Horizonte, Brazil

**Keywords:** cancer, chemotherapy, liposome, triggered release, tumor environment

## Abstract

Since more than 40 years liposomes have being extensively studied for their potential as carriers of anticancer drugs. The basic principle behind their use for cancer treatment consists on the idea that they can take advantage of the leaky vasculature and poor lymphatic drainage present at the tumor tissue, passively accumulating in this region. Aiming to further improve their efficacy, different strategies have been employed such as PEGlation, which enables longer circulation times, or the attachment of ligands to liposomal surface for active targeting of cancer cells. A great challenge for drug delivery to cancer treatment now, is the possibility to trigger release from nanosystems at the tumor site, providing efficacious levels of drug in the tumor. Different strategies have been proposed to exploit the outer and inner tumor environment for triggering drug release from liposomes and are the focus of this review.

## Introduction

A liposome is a spherical vesicle composed of a phospholipid bilayer formed into an enclosed aqueous pocket. They were first described by Alec Bangham in the mid-60s, who was interested in the system as an *in vitro* model of biological membranes (1). It was Gregory Gregoriadis in 1973, who first proposed their use as a drug delivery system (2). As drug carriers, liposomes are extremely versatile, as the phospholipid bilayer can accommodate hydrophobic drugs, while hydrophilic drugs can be entrapped on the aqueous inner compartment. In 1974, Gregoriadis et al. first suggested the potential of liposomes as carriers of anticancer drugs, based on the observation that they were able to accumulate in the tumors (3). This accumulation ability was later explained by the idea that liposomes take advantage of the leaky vasculature and poor lymphatic drainage present at tumor tissue to passively accumulate in this region, what is known as Enhanced Permeability Retention (EPR) effect (4–6). A major drawback of the first generation liposomes consisted on its rapid uptake by the mononuclear phagocyte system (MPS) after systemic administration, which limited their application. It was in 1990 that Klibanov et al. reported the first step on liposome’s evolution: the possibility of enhancing circulation time by coating the liposomal surface with inert biocompatible polymers, such as polyethylene glycol (PEG). This coat prevents the recognition of liposomes by opsonins and thus reduces their clearance by the cells of the MPS (7). As the EPR effect is a progressive phenomenon, requiring many passages of the nanosystem through tumor vasculature, long circulating liposomes have a better chance to accumulate in tumors (5). The strategy of coating liposomes with PEG allowed for the development of Doxil^®^, the first FDA-approved nano-drug (8, 9).

Aiming on further improving tumor target, research efforts have been made to develop liposomes able to actively target the tumor site. The strategy consists on attaching a ligand to the liposome surface, directed to a molecule or receptor over expressed on the tumor cell. This strategy is also complementary to EPR effect as actively-targeted liposomes require being in the vicinity of their target to recognize and interact with it. No actively targeted liposomal formulation is commercially available; however, some have made it to the clinical development stages (10).

**Figure 1:**
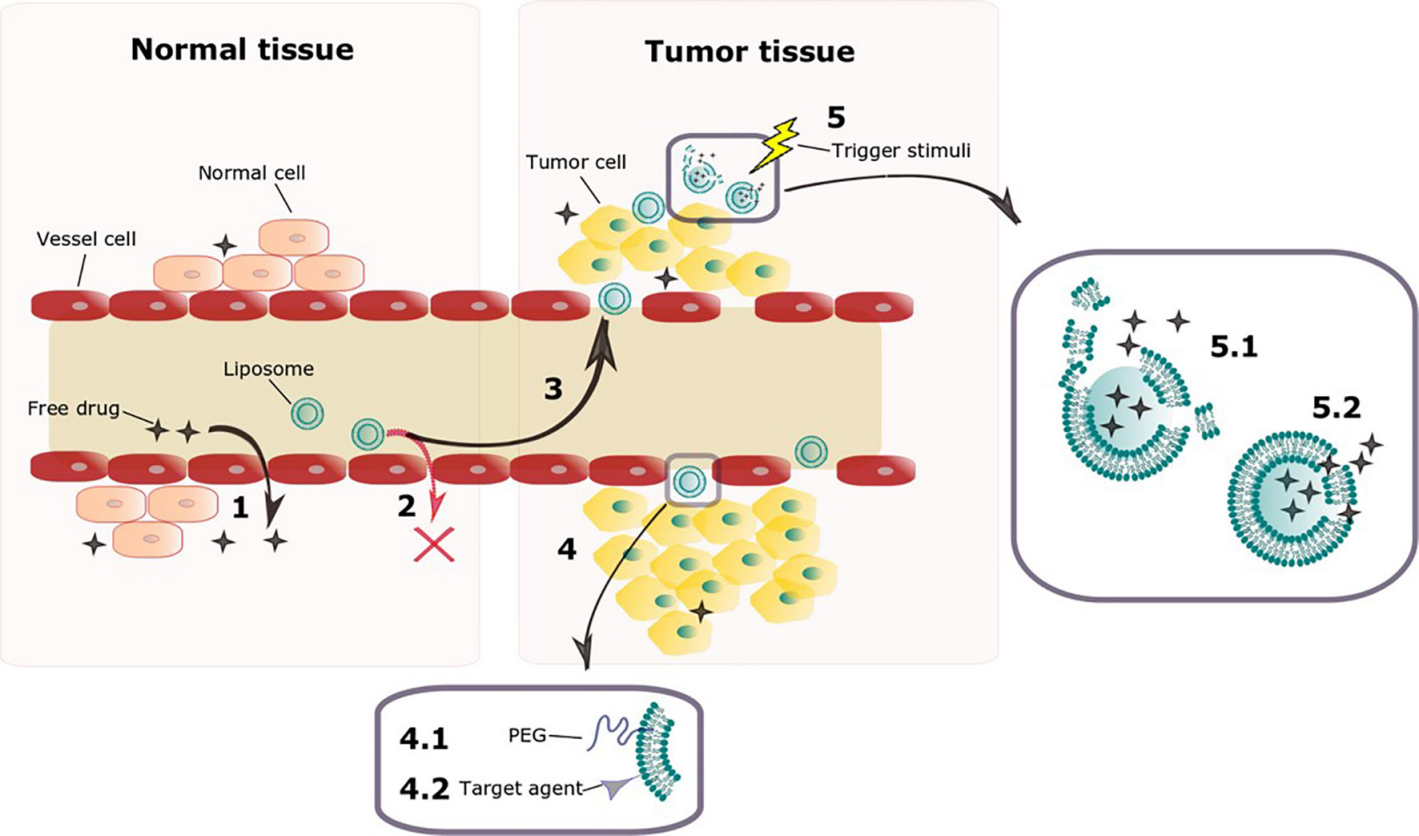
Schematic representation of key features of liposomes as carriers of anticancer drugs and its evolution. The tight endothelial junctions of cells on normal vessels allow free drug to overflow from blood vessel to normal tissue (1). Liposomes however are too big to penetrate through the tight junctions on vessels from normal tissues (2), accumulating preferably on tumor tissue, where vessels present a defective architecture (3) on a process known as EPR effect. Liposomal surface functionalization can be done aiming on improving its characteristics (4). Surface PEGylation enhances circulation time so that EPR effect is enhanced (4.1). Attaching targeting ligands allows liposomes to recognize tumor cells once they leak through the vasculature (4.2). Once on tumor tissue, different endogenous and exogenous trigger stimuli are being investigated as possibilities to improve drug release on this site (5). A trigger stimulus can either disrupt completely the liposomal membrane (5.1) or enhance its permeability (5.2) allowing the drug to escape.

For both targeted and non-targeted liposomes, drug release kinetics is critical to the anticancer effects, thus a great challenge now facing drug delivery for cancer treatment is liposomal trigger at tumor site. Liposomes designed upon this concept should be optimized to prevent drug release in the bloodstream and normal tissues and release their contents only when exposed to a trigger stimulus at tumor site, obtaining optimum anticancer effects (11, 12). This strategy aims on enhancing efficacy and reducing toxicity, is highly dependent on EPR effect, and can also benefit from active target. **Figure 1** exemplifies the idea of liposomes as carriers of anticancer drugs and its evolution.

Different strategies have been proposed to exploit the outer and inner tumor environment for triggering drug release from liposomes. Formulations designed to be triggered by extracorporeal physical stimuli include thermo-, magnetic-, ultrasound-, light-, chemically- and electric-triggered liposomes (13). Physiological signals present at the tumor microenvironment, such as the presence of acidic pH, redox potential (glutathione, GSH), enzymes, hypoxia, and adenosine-50- triphosphate (ATP) have been explored as endogenous trigger stimuli (14). Compared to endogenous triggers, exogenous triggers have the advantage of being much more controllable. It is not only possible to better control when the treatment occurs and its duration but additionally there are less inter-patient variations as for the endogenous signals (14). To date the only formulation planned according to this concept to reach clinical trials is Thermodox^®^, thermo-sensitive liposomes currently in phase III trial (15). The different liposomal trigger strategies will be further discussed herein. It is beyond the scope of this review to make an exhaustive list of the formulations developed to date. On the contrary, we aim to give key examples of formulations built upon each of these strategies, providing an overview of the state of the art on possible liposomal compositions and trigger mechanisms.

## Triggered Drug Release by Exogenous Stimuli

### Thermo-Triggered Liposomes

#### Thermal Therapy and Thermo Sensitive Liposomes

The earliest historical evidence of the use of thermal therapy (or hyperthermia) as cancer treatment was recorded by the Egyptian Edwin Smith Surgical Papyrus in 3000 B.C (16). The role of thermal therapy on cancer management is based on the higher sensitivity of cancer cells to temperature oscillations when compared to normal cells (17–19). Different heating modalities such as radiofrequency (RF), ultrasound transducers, laser and microwave based methods have been exploited to deliver local hyperthermia treatment to deeply seated tumors (16). High temperatures not only cause direct injury to cancer cells but also sensitize them to other treatment modalities, such as chemotherapy and radiotherapy (18). Concerning the combination with chemotherapy, the strategy is further improved when the drugs are encapsulated in nanosystems, as mild hyperthermia (39–42°C for ~ 60 min) can induce several effects on tissues. These effects include increased blood flow, improved perfusion, enhanced oxygenation and increased permeability, which enhances EPR effect, favoring the extravasation of the nanosystems into the tumor. Additionally, mild hyperthermia can be the trigger for a site specific release of the drug from thermosensitive nanosystems (16, 20). Different nanosystems such as polymeric micelles, hydrogels and dendrimers have been described as thermosensitive, however, liposomes are the most successful example of this concept to date (21). A thermosensitive liposome (TSL) formulation, ThermoDox^®^, reached Phase III clinical trials (15). For TSL to be effective, two main requirements must be fulfilled: minimum leakage of the encapsulated drug under physiological temperature (37°C), and release of the encapsulated drug under mild hyperthermia (22). Knowing that drugs are released from TSL at the melting phase transition temperature (Tm) of the lipid bilayer, these requirements can be fulfilled by taking advantage of the physical properties of liposomal membranes. At Tm, the structure of the lipid bilayer changes, as a transfer from a solid gel phase (Lβ) to a liquid-crystalline phase occurs (Lα). This results in an increased permeability of the lipid bilayer to its aqueous contents *via* passive transfer through the disordered membrane phase boundaries. Thus, TSL have been designed with transition temperatures around 40–42°C, so that the leakage is minimized at body temperature but content is rapidly released when liposome passes through a tumor heated to a temperature around Tm (20, 23).

#### The First Steps on Thermo Sensitive Liposomes Development

**Figure 2:**
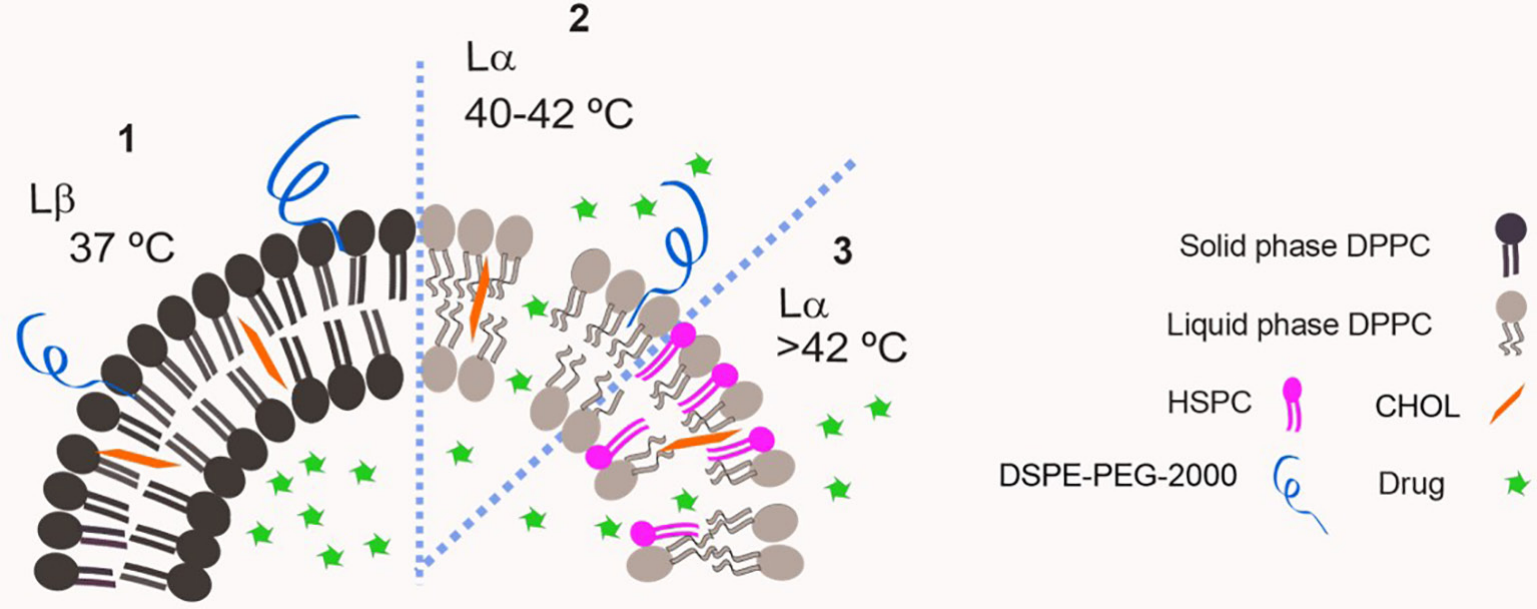
Schematic representation of a classic thermo-sensitive liposome. DPPC has a Tm= 41°C therefore it is in solid phase at 37°C and bilayer permeability is low (1). Increasing temperature above Tm leads to high bilayer permeability and drug leakage as DPPC is in liquid phase (2). The insertion of a lipid with higher Tm on the bilayer e.g. HSPC, Tm=52°C, allows the modulation of transition temperature of the membrane (3).

In 1978, Yatvin et al. described the first TSL, composed of dipalmitoyl phosphatidylcholine (DPPC, Tm= 41°C) and distearoyl phosphatidylcholine (DSPC, Tm = 54°C) at a 3:1 molar ratio. They demonstrated that the ratio of release of the contents from these liposomes at 44°C to that at 37°C could be made greater than 100:1 in the presence of fetal bovine serum. That drew attention for the possible applications of these systems for cancer treatment (24). On the next year, Weinstein et al. (25) published the results showing that a formulation composed of DPPC : DSPC (7:3 weight ratio) encapsulating methotrexate delivered more than four times as much the drug to murine tumors heated to 42°C compared to unheated control tumors. This formulation however, was largely cleared from circulation in 1 h (25). Since then, various modified compositions were proposed for TSL, aiming to increase stability at body temperature (reducing leakage) and enhancing the blood circulation time. Gaber et al. (26) evaluated a series of liposomes composed of DPPC, hydrogenated soybean phosphatidylcholine (HSPC, Tm=52°C), cholesterol (CHOL) and 1,2-distearoyl-sn-glycero-3-phosphoethanolamine-N-[methoxy(polyethylene glycol)-2000] (DSPE-PEG2000) aiming on optimizing the thermo-sensitivity and circulation time of TSL, as illustrated in **Figure 2**. They reported that the presence of CHOL is important to stabilize liposomes containing DSPE-PEG2000 on human plasma, however if CHOL concentrations are above 30% the phase transition is avoided and thermo-sensitivity is lost (26). They defined the optimal formulation as DPPC : HSPC : CHOL : DSPE-PEG2000 (50:25:15:3 molar ratio), which was then used to encapsulate DXR and tested *in vivo* on a model of mammary adenocarcinoma in rat skin. At the skin temperature (34°C) a negligible release was observed, and it increased 38 and 76-fold when skin temperature was raised to 42 and 45°C, respectively, for 1 h (26).

#### Lysolipid-Containing Thermo Sensitive Liposomes

**Figure 3:**
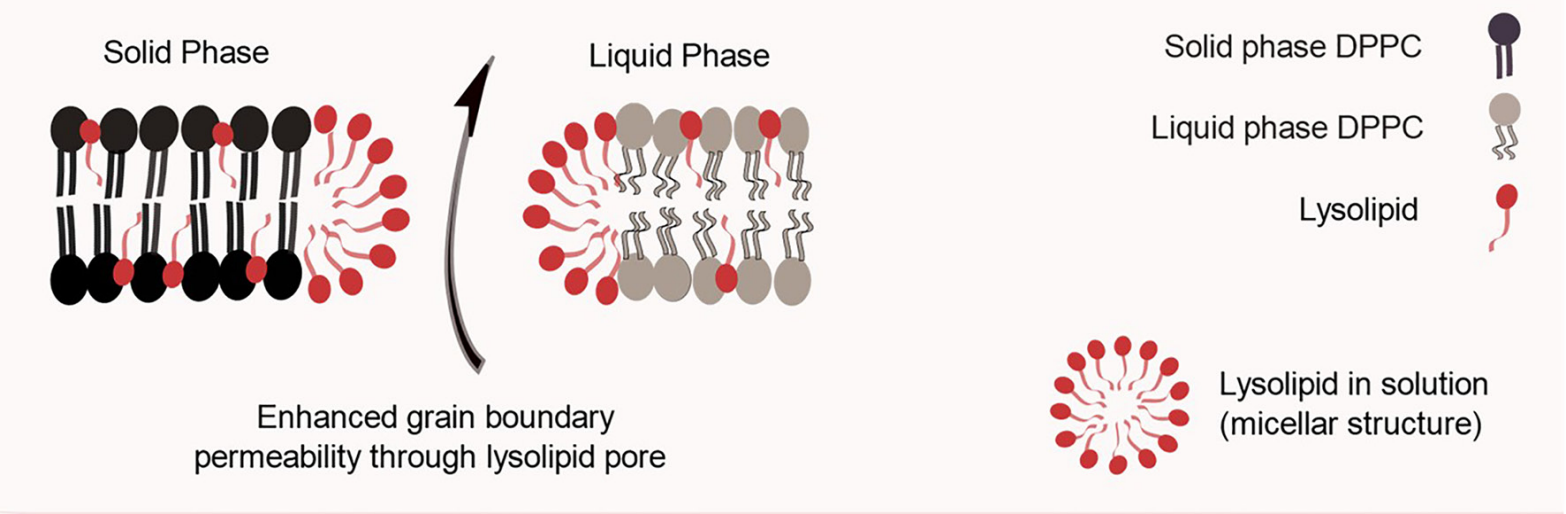
Schematic representation of a lysolipid containing thermo-sensitive liposomal bilayer. One hypothesis for lysolipid triggering mechanism is based on its tendency to form micelles, which leads to nanopores in grain boundary regions.

In 2000, Needham et al. reported the results for a lysolipid-containing TSL, which is considered the major breakthrough on the field to date. The obtained liposomes were composed of DPPC:1-myristoyl-2-palmitoyl-sn-glycero-3-phosphocholine (MPPC):DSPE-PEG2000 (90:10:4 molar ratio) (11). The presence of a few mol% of the MPPC lysolipid in the bilayer leads to a dramatically enhanced grain boundary permeabilization, possibly due to the formation of nanopores in these regions, as illustrated in **Figure 3** (27). That allows for a rapid release of liposome contents in response to a heat stimulus within the mild, clinically-achievable hyperthermia range of 40–42°C. These liposomes were used to encapsulate DXR, and released 45% of its contents in 20 s when exposed to 42°C. As means of comparison, they prepared a formulation composed of DPPC : HSPC : CHOL : DSPE-PEG2000 (50:25:15:3 molar ratio) as described previously by Gaber et al. and as expected this formulation took 30 min to release 40% of its content at 42°C. They also prepared a non-thermo-sensitive liposomal formulation composed of HSPC : CHOL : DSPE-PEG2000 (75:50:3 molar ratio), which did not release any drug upon heating to 42°C. When tested for its antitumor efficacy in a human squamous cell (FaDu) carcinoma xenograft, the lysolipid-containing TSL (100% of animals presented tumor local control (LC) defined as no tumor present at 60 days after treatment). It was significantly more effective than non-thermo-sensitive liposomal formulation (0% LC) or the formulation described by Gaber et al. (10% LC), thus showing the importance of enhanced drug release for achieving the best antitumor efficacy (11). Other preclinical studies using different mice models and a Phase I trial with dogs presenting spontaneous tumors were performed confirming the potential of the formulation (28, 29). The encouraging tumor responses supported clinical evaluation of this formulation. For that, it was slightly modified to DPPC: 1-myristoyl-2-stearoyl-sn-glycero-3-phosphocholine (MSPC):DSPE-PEG2000 (86:10:4 molar ratio) and so called ThermoDox^®^.

To date, ThermoDox^®^ has predominantly been used clinically in conjunction with radiofrequency ablation (RFA). The idea consists on achieving tumor core ablation with RFA as DXR is intended to improve therapy of the tumor borders (30). A phase I study revealed that ThermoDox^®^ could be safely administered systemically at its maximum tolerated dose (MTD) (50 mg/m^2^) in combination with RFA, with limited and manageable toxicity (31). The combination treatment moved directly into a Phase III evaluation (HEAT trial) which included 701 patients with hepatocellular carcinoma (HCC) in 79 clinical sites in 11 countries. In 2013 it was announced that this study did not meet its primary endpoint of a 33% improvement in progression-free survival (PFS) compared to RFA alone. A post-hoc subgroup analysis however, demonstrated a 53% risk improvement in overall survival (OS) in the subset of patients that received optimized RFA treatment for 45 min or more combined with ThermoDox^®^, compared to RFA alone (15, 30). Another phase III clinical study of ThermoDox^®^, so called “OPTIMA trial”, was initiated standardizing the RFA to 45 min or more for patients with HCC. It was initiated in June 2014 and is estimated to enroll about 550 patients. The primary endpoint is OS with PFS as a secondary endpoint. The “TARDOX trial” is a phase I clinical trial aiming on evaluating the feasibility of the combination of ThermoDox^®^ with focused ultrasound (FUS) for treatment of patients with liver tumors. Unlike RFA, FUS is a non-invasive clinical treatment modality. ThermoDox^®^ is also been evaluated for the treatment of other solid tumors. In the “DIGNITY trial”, ThermoDox^®^ is being investigated for the treatment of chest wall cancer under superficial hyperthermia (30). There are also Phase I studies to evaluate its combination with local hyperthermia for patients with breast cancer and with high-intensity focused ultrasound guided by magnetic resonance for pediatric refractory solid tumors (32, 33).

As promising components of TSL, other lysolipids continue to be investigated. Lyu et al. (35) described liposomes containing the lysolipid 1-stearoyl-2-hydroxy-sn-glycerol-3-phosphocholine (1-StePc) for delivering Marimastat (MATT), a matrix metalloproteinases (MMPs) inhibitor, to the tumor microenvironment. For evaluating the *in vitro* drug release abilities, liposomes composed of DPPC:1-StePc : DSPE-PEG2000 (86:10:4 weight ratio) encapsulating (6)-carboxyfluorescein (CF) were analyzed for its release profile. After 90 min, fluorescence intensity for liposomes kept at 43°C was around nine times higher when compared to that observed for liposomes kept at 39°C. When tested *in vivo* for its antitumor activity, animals receiving the MATT-TSL with applied hyperthermia showed 15-fold tumor growth, contrasting with the observed 35-fold growth for animals on control group receiving saline. Regarding the metastatic foci, animals receiving the MATT-TSL plus hyperthermia presented a 7-fold decrease in metastatic lung foci compared to animals on control group. Unfortunately authors did not include groups treated with MATT-TSL only, without hyperthermia, or a treatment with non-TSL liposomes encapsulating MATT for means of comparison (34).

#### Other Strategies for Thermo Sensitive Liposomes Development

Other strategies for improving TSL have been described. One of these strategies is the use of 1,2-dipalmitoyl-sn-glycero-phosphodiglycerol (DPPG2, Tm = 39.7°C). This phospholipid allows for an increase in the circulation half-life of vesicles with the advantage that it can be used in concentrations up to 70% mol, against around 10% of PEGylated lipids, which act like surfactants in high concentrations (35). A simplified TSL was developed by Tagami et al. (36). This formulation is composed of DPPC : Brij78 (96:4 molar ratio). Brij78 is a non-ionic surfactant consisting of a PEGylated acylchain and was evaluated as a substitute for the MSPC lysolipid. They reported a 1.4-fold increase in drug delivery to the locally heated tumor (~43°C) and enhanced tumor regression for the new liposomes compared to ThermoDox^®^ (36).

Another strategy is the conjugation of elastin-like polypeptides (ELPs; either in N-terminus or in lysine residues) to liposomes containing N- hydroxysuccinimide (NHS) groups in their bilayer. ELPs are thermo-responsive protein-based biopolymers based on the amino acid sequence of elastin. They are thermo sensitive going an inverse temperature transition (ITT) in response to temperature change, which means they are soluble in aqueous solution at temperatures below their ITT, but above their ITT they are insoluble and aggregate (37).

Choi et al. (38) obtained liposomes composed of HSPC : CHOL : DSPE-PEG-NHS (75: 50: 3 molar ratio) and conjugated different ELPs to the liposome surface. Single conjugated ELP liposomes were obtained by using ELPs with only one amino group at each N-terminus and multiple conjugated ELP liposomes were obtained when ELPs containing internal lysine residues were used. They could show that above the transition temperature of the ELPs, they aggregate and distort the liposome membrane resulting in a crack followed by drug release. The extension and speed of drug release was shown to be closely dependent on the conjugation manner and also on the length of the ELP (38).

### Magnetically Triggered Liposomes

The biomedical applications of magnetic fields involve both diagnostic and therapeutic applications. Magnetic resonance imaging (MRI) is widely used on cancer management, as it plays a pivotal role in diagnosis, staging and monitoring treatment efficacy for certain tumors (39). Paramagnetic and superparamagnetic nanoparticles enhance MRI specificity and can be obtained from different materials, such as metals (gold, silver, and cobalt) or metal oxides (Fe_3_O_4_, TiO_2_, and SiO_2_). By now, iron oxides have been the most widely used in the clinics (40). Magnetic nanosystems are also widely investigated for tumor targeting and hyperthermal therapy. The first consists on applying an external magnetic field to surface tumors, what should attract and maintain magnetic nanoparticles (MN) into this area. Hyperthermal therapy consists on the exposure of MN to an alternating magnetic field, allowing for a local temperature rise and tumor cell kill (41–44). The magnetic-trigger of nanosystems is another promising strategy. Among the different available options for external stimulus, magnetism is considered as one of the best, as almost no physical interaction with the body occurs when comparing to light irradiation, ultrasound or electrical fields as stimuli (45).

Different nanosystems having a magnetic material on their composition have shown to be triggered by applying an alternating magnetic field, such as polymeric nanosystems, nanoparticles, micelles and liposomes (46–57). Most of the times, the strategy consists on combining thermo to magnetically trigger. Once magnetic components are added to thermoresponsive carriers, they can generate heat in response to a high-frequency (in the range of hundreds of kilohertz or higher) alternating current magnetic field (ACMF). Once this generated heat raises the temperature of the liposome’s membrane around the Tm, its permeability is greatly enhanced and the cargo can thus be release (54). This strategy is superior to those for thermo-trigger alone as ACMF penetrates deep into the tissue, so that it reaches the magnetic nanosystems to generate a localized heat without damaging normal hypodermal tissues (48, 50). Another possibility is still the mechanical deformation of a nanocarrier when submitted to a low-frequency ACMF. This acts as the immediate cause of drug release and is of practical importance for cases on which hyperthermia might be detrimental (13, 56).

Nanosystems containing iron oxides, particularly magnetite (Fe3O4), maghemite (γ-Fe2O3) and ferrites (mixed oxides of iron and other transition metals) are the most promising because of their low toxicity and easy clearance, heat generation ability and chemical stability (13, 50). Different coatings of iron oxides allow the MN to present different water solubilities. Liposomes herein presented were all obtained by thin-film hydration method. On different formulations, hydrophobic iron oxide MN were added directly to the lipids during film formation (54, 57) and hydrophilic iron oxide MN were added during the film hydration (52, 53, 55, 56). Theoretical studies suggest a threshold maximum nanoparticle diameter of 6.5 nm for incorporation of neutral nanoparticles into lipid bilayer (54).The MN used on different formulations herein presented had mean diameter ranging from 3 to 10 nm.

#### Magnetic Trigger Using Thermosensitive Liposomal Bilayers

Different *in vitro* release studies have demonstrated that the submission of liposomes encapsulating a MN to ACMF allows an augmented content release. Tai et al. (52) developed a formulation composed of DPPC : CHOL (5:1 weight ratio) encapsulating dextran-coated iron oxide nanoparticles (3–5 nm) and a fluorescent tracing molecule, CF. This formulation was able to release the whole CF content in around 3 min when exposed to ACMF, while liposomes without the MN led to no significant CF release under the same conditions (52). Amstad et al. (54) obtained liposomes composed of DSPC : DSPE-PEG2000 (10:0.5 molar ratio) encapsulating palmityl-nitro DOPA-stabilized iron oxide MN (5-10 nM) and the self-quenching dye calcein. An increase in fluorescence directly translates into release of calcein, and the fluorescence of liposomes lacking MN did not change upon ACMF treatment, while an increase of around 270% on the fluorescence of liposomes containing the MN in their membrane when submitted to an ACMF was observed. In this study, they evaluated the liposomes after exposure to ACMF, observing that it did not affect the hydrodynamic diameter of the vesicles loaded with MN. Also, no precipitation of MN was observed after ACMF treatment, demonstrating that the liposomes remained intact and at constant size during this treatment. It was possible to conclude that the calcein release was due to a change in membrane permeability and not their rupture or fusion, what allows for the content to be repeatedly and nondestructively released (54). Hardiansyah et al. (56) obtained liposomes composed of HSPC : DSPE : CHOL (12.5:1:8.25 mole ratios) encapsulating citric acid-coated iron oxide MN (10 nm) and doxorubicin (DXR). Liposomes encapsulating both MN and DXR presented 80% DXR release in the end of 10 min when exposed to ACMF against around 50% for non-magnetic liposomes (55). Hardiansyah et al. (57) obtained liposomes composed of DPPC : CHOL : DSPE-PEG2000 (80:20:5 molar ratio). Cumulative curcumin release during 30 min from liposomes submitted to ACMF treatment was 15 and 2.7-fold higher than that observed for liposomes incubated at 37 and 45°C, respectively, without ACMF treatment (57).

Pradhan et al. (53) designed liposomes that are both magneto-thermosensitive and actively targeted to folate receptors. These liposomes were composed of DPPC : CHOL : DSPE-PEG2000:DSPE-PEG2000-Folate (80:20:4.5:0.5 molar ratio) encapsulating DXR and MN (FluidMag-HS, 10 nM). The thermosensitivity on DXR release at 43°C was demonstrated as 53% of DXR content was released at this temperature against 17% at 37°C. When exposed to a permanent magnetic field *in vitro* aiming to physically target cell lines expressing folate receptor (KB and Hela cell lines), higher uptake was observed for the formulation compared to commercially available liposomal DXR, non-magnetic folate-targeted liposomes and free DXR, which resulted in superior cytotoxicity. Magnetic hyperthermia at 42.5°C and 43.5°C further increased the cytotoxicity of the obtained liposomes, which was attributed to the superior release of DXR triggered by heat generated by the MN (53).

#### Magnetic Trigger Using Non-Thermosensitive Liposomal Bilayers

Avoiding hyperthermia generation has potential application for some cancers where temperature changes are detrimental, such as brain cancers. Based on this fact, Guo et al. (56) designed a magnetic formulation that is not thermosensitive, and that does not imply any increase of temperature. These liposomes were composed of phosphatidyl-choline (PC):CHOL:amphiphilic carboxymethyl dextran (CMD) (55:40:0.5 molar ratio) encapsulating iron oxide MN and DXR. Exposure of these liposomes to a low-frequency ACFM at pH 5.0 allowed for 74% of DXR release while in 24h incubation only 35% release was observed for liposomes that were not submitted to ACFM. When influence of ACFM on liposomal structure was investigated, it was observed that they became much larger after the exposure to ACFM. Thus, the initial drug release from the liposomes under ACFM was attributed to the drug leakage resulting from the liposomal structure deformation (56).

### Ultrasound-Triggered Liposomes

Ultrasounds (US) are mechanical longitudinal waves with a periodic vibration in frequencies superior to human audible range (20 kHz) that propagate due to pressure changes in the medium (58, 59). This technology is already widely used in the medical field for diagnosis and therapeutic purposes due to non-invasiveness, safety and cost effectiveness.

However, some disadvantages can be pointed out, such as cavitation skin burns due to the presence of air between the transducer and the body surface. This also limits the treatment of extensive superficial regions, such as breast cancer, and regions where air is inherently present, such as lungs and intestines. The presence of obstacles such as bones in the proximity of the organ under treatment also complicates access to the region. Yet another challenge is to focus on organs that have movement (59, 60).

US can be used to trigger liposomes. Different mechanisms might explain the trigger by US, such as cavitation, acoustic streaming and hyperthermia, and most probably they are not independent (61).

In acoustic cavitation, there is the interaction of acoustic waves with gas bubbles. On stable cavitation, bubbles oscillate around an equilibrium radius causing fluids to flow around the bubbles. As acoustic pressure increases, the process known as inertial cavitation takes place, in which the gas bubbles undergo rapid growth and violent collapse upon US exposure. This creates high pressures and increases the local temperature, inducing thermal dissociation of water and, therefore, the formation of reactive oxygen species (ROS). Acoustic streaming such as microstreaming is another effect of cavitation that can induce shear stresses that can destabilize liposomes and permeabilize cell membranes (58, 60, 61).

The hyperthermia caused by US is associated to the absorption of the ultrasonic waves by the tissue, creating mechanical compression and decompression. Some of this mechanical energy is lost due to friction effects and converted into heat. The composition of the lipid bilayer of the liposomes is known to play a role on their US sensitivity, increasing their content release. Liposomes with thermo-sensitive composition, as previously described, facilitate the release of drugs by US induced hyperthermia. The presence of PEG also contributes to this function since it absorbs the energy of the ultrasound by concentrating it on the surface of the vesicle (60).

TSL composed of DPPC: MPPC: DSPE-PEG2000: DSPE-PEG2000-iRGD (86:10:2:2 molar ratio) encapsulating DXR (iRGD-LTSL-DXR) were obtained by Deng et al. (62). iRGD peptide, like conventional RGD peptides, target tumors by binding to αv integrins selectively overexpressed on the tumor angiogenic endothelial cells as well as tumor cells. When iRGD-LTSL-DXR was administered to mice bearing 4T1 breast cancer tumors, to explore the anti-tumor effects in combination with high intensity focused ultrasound (HIFU) they observed delayed tumor growth after a single-dose treatment (at a DXR equivalent of 5 mg/kg). The inhibition of iRGD-LTSL-DXR + HIFU were 65.2 ± 6.1% (p < 0.001), while without HIFU, the tumor inhibition rate of iRGD-LTSL-DXR was only 33.1 ± 7.6% (62).

Vanosdol et al. (63) prepared TSL composed of DPPC: MSPC : DSPE-mPEG2000 (85.3: 9.7: 5.0 molar ratio) encapsulating DXR. In order to take advantage of cavitation, they also prepared this formulation with incorporated perfluoropentane gas (PFP5). *In vivo* biodistribution studies showed that when tumors were submitted to high intensity focused ultrasound (HIFU) reaching 42°C, DXR accumulation was higher compared to that observed for tumors at body temperature (37°C). For the non-PFP5 formulation, ~0.9 μg DXR/gram of tissue was observed for non-HIFU treated tumors while ~3.8 μg DXR/gram of tissue was observed for HIFU treated tumors. For the formulation encapsulating PFP, ~2.1 μg DXR/gram of tissue was observed for non-HIFU treated tumors while ~5.1 μg DXR/gram of tissue was observed for HIFU treated tumors. This approximate 1.4-fold greater drug delivery observed for the PFP5 containing formulation at 42°C indicated that the additive response of cavitation to HIFU treatment (63).

For mitochondria targeted sonodynamic therapy (SDT), using ultrasound and a sonosensitizing chemical substance, Chen et al. (64) developed a liposomal formulation composed of soy lecithin:CHOL : CHOL-anchored 3-carboxypropyl triphenylphosphine bromide (3:0.7:0.3 weight ratio). This formulation was used to encapsulate the sonosensitizer hematoporphyrin monomethyl ether (HMME) (64). The presence of CHOL-anchored 3-carboxypropyl triphenylphosphine bromide (CHOL-TPP) allows for mitochondria targeting. Because of their role in regulating key cellular functions, mitochondrial targeting compounds represent a promising approach to eradicate cancer cells that are refractory to chemotherapy. The release of HMME from liposomes could be triggered by ultrasound due to the oxidation of the lipid in liposomes. After incubation with cancer cells, the TPP modified liposomes (Lipo-TPP) could accumulate in the mitochondria and assist the HMME to achieve greater cancer cell inhibition effect in SDT. *In vitro* cytotoxicity studies of liposomes containing the sonosensitizer HMME and the mitochondrial target TPP followed by ultrasound for 3 min (HMME-Lipo-TPP + SDT) against MCF-7 cells was evaluated. This study indicated that the cell viability for the HMME-Lipo-TPP + SDT treatment was approximately 35%. On the other hand, cell viability around 75% was observed for cells treated with liposomes containing only TPP (Lipo-TPP + SDT), showing that there was a significant increase in cytotoxicity when using the sonosensitizer associated with ultrasound (64).

In 2016, Ninomiya et al. obtained liposomes composed of 1,2-dimyristoyl-sn-glycero-3-phosphatidic acid (DMPA), DPPC and CHOL (1:4:5 molar ratio). These liposomes were modified with avidin, which has an affinity for cancer cells and the envelope of the hemagglutinating virus of Japan (HVJ), which promotes the fusion of liposomes to cells and then used to encapsulate perfluoropentane nanoemulsion PFC5. The PFC5 liquid has a low boiling, and the nanoemulsion droplets are converted into much larger gas bubbles by ultrasonic induced droplet aporization (ADV). These bubbles elongate and disrupture the liposome membrane, allowing for the encapsulated drug to be released. The increased US-mediated disruption of liposomes encapsulating PFC5 was confirmed on an assay evaluating the optical density at a wavelength of 600 nm (OD_600_). After US irradiation, liposomes without PFC5 presented a relative OD_600_ value of 92.7% of that without US irradiation, indicating no significant disruption of the liposomes by US. On the other hand, for PFC5-loaded liposomes, the turbidity of the suspension decreased after US irradiation with a relative OD_600_ of only 9.5% of that without US irradiation. To investigate cancer cell injuries mediated by this formulation, MCF-7 cells (human breast cancer) were treated and either submitted to US irradiation or not. Cell viability was then determined. Cells receiving the formulation followed by US irradiation had their viability reduced to 43%, whereas cells receiving treatment with the formulation in the absence of US showed cell viability equal to 80%. Cell viability of those which received US-irradiation alone was about 80%, confirming that the US potentializes the formulation (65).

### Light-Triggered Liposomes

The use of light stimuli to trigger the controlled release of drugs from liposomes has been studied as the wavelength, energy intensity and time of exposure and beam diameter are adjustable with high precision, which may aid in individual pharmacotherapy (64, 66, 67). Photodynamic therapy is based on the local or systemic application of a photosensitive compound - the photosensitizer, which is accumulated in pathological tissues. Photosensitizing molecules absorb light from the appropriate wavelength, initiating activation processes leading to the selective destruction of inappropriate cells. Photo cytotoxic reactions occur only within the pathological tissues, in the area of distribution of the photosensitizer, allowing the selective destruction (68) Photosensitizing agents, when added to liposomes, can generate ROS, like singlet oxygen, upon excitation by light at specific wavelengths. This singlet oxygen consequently induces oxidative stress, rupture of the membranes and formation of pores allowing the contents of the vesicle to escape (69–71)

Based on photodynamic therapy Luo et al. (72) developed liposomes composed of DSPC: Dioleoylphosphatidylcholine (DOPC): CHOL: Porphyrin-phospholipid (PoP) containing encapsulated DXR. The presence of the unsaturated lipid DOPC accelerates the DXR release by oxidation mechanisms of this lipid. The inclusion of the photosensitizer POP would enhance this release. Thus, the team evaluated different concentrations of DOPC, between 0 and 10 moles, and also POP. It has been proven by mass spectrometry that DOPC accelerates the release of DXR and that in the presence of an oxygen scavenger or an antioxidant, the release of the drug is inhibited, suggesting the mechanism of release by oxidation. The inclusion of increasing amounts of DOPC accelerated and increased DXR release. At 5 mole% DOPC and 0.3 mole% POP, a 50% DXR release was observed in 43 s. Higher amounts of DOPC led to destabilization of the vesicles in the absence of light. Therefore, the final formulation of work developed was composed of DSPC: DOPC: CHOL: PoP, in the 54.7: 5: 40: 0.3 molar ratios respectively (72).

In another moment, Luo et al. (73), encapsulated DXR in a similar liposomal formulation composed of DSPC: CHOL: DSPE -PEG: PoP, (53:40:5:2 molar ratio). This time, DSPE-PEG was used to obtain stealth liposomes to treat human pancreatic adenocarcinoma. *In vivo* drug release was triggered by the oxidation of DOPC and CHOL after exposure to 665 nm NIR. Tumor uptake of DXR was assessed and shown to be 7-fold higher for tumors of animals receiving PoP-DXR followed by laser exposure when compared to treated animals without laser exposure. An inhibition of tumor growth *in vivo* demonstrated excellent chemotherapy efficacy. Treatment with PoP-DXR (DXR=7mg/Kg) led to regression of tumor volume to values below 20 mm^3^ after 2 weeks of treatment. In animals treated with liposomes without PoP, therefore not light triggered, tumors evolved up to 500 mm^3^. Animals treated with PoP-DXR survived until the end of the study, 60 days, while animals receiving the same treatment but without laser stimulation died after 40 days of treatment due to disease progression (73).

Following the same principle, in a study by Fuse et al. (74) liposomes co-encapsulating the photosensitizer talaporfin sodium (TPS) and the drug gemcitabine (GEM) were evaluated for their cytotoxic activity against EMT6/P breast cancer cells. Liposomes were composed of DSPC: DOPE: CHOL: DSPE-PEG2000 (85:10:5:5 molar ratio). Cells receiving NIR laser irradiation after incubation with the formulation had cell viability lower than 5%, In contrast, around 90% cell viability was observed for cells exposed to the formulation only, without NIR laser irradiation (74).

Also based on photodynamic therapy, Li et al. (75) developed NIR sensitive liposomes for breast cancer treatment. A phospholipid material of special structure 1- (1z-octadecenyl) -2-oleoyl-sn-glycero-3-phosphocholine (PLsPC) and a hydrophobically modified fotosensibilizer Indocyanine green and octadecylamine (ICG-ODA) were employed for liposome light-sensitivity (LSL). The other used lipids were CHOL and DSPE-PEG2000. DXR was encapsulated in the liposome which surface was subsequently conjugated with Her2 antibodies and obtained the unique nanosystem Her2-I & D-LSL. The encapsulation efficiency and stability were closely related to the ratio of S100 and PLsPC and the proportion of ICG-ODA. The formulation S100: CHOL = 5:1, S100∶PLsPC = 4:1 or 2:1 and ICG-ODA: total lipid = 1:10, with a high EE % and low DXR leakage was chose to proceed the study. *In vitro* cytotoxicity of this formulation followed by NIR laser irradiation was evaluated against MCF-7 (human breast cancer) cell line. After treatment with Her2-I & D-LSL combined to laser, there was almost 100% cell death, against over 60% of death for cells treated with Her2-I & D-LSL without laser irradiation. The same was evaluated for a non- HER2 targeted formulation. Cytotoxicity was approximately 65% for cells that received this treatment followed by laser versus only 40% for cells that received the treatment in the absence of the NIR (75).

*In vivo* antitumor was evaluated in mice bearing MCF-7 tumors. A significant difference in the volume and weight of tumors of animals exposed to both Her2-I & D-LSL and laser compared to those treated with the formulation only was observed. After 30 days of Her2-I & D-LSL plus laser treatment, tumors had a weight of approximately 25 mg while tumors of animals treated in the absence of the NIR had an approximate weight of 150 mg. For animals with SKOV-3 cell tumors a similar pattern was observed. After treatment with Her2-I & D-LSL plus laser, the tumor showed almost total regression whereas the treatment without NIR allowed the tumor to reach a weight close to 0.4 g (75).

Making use of photosensitizers to generate ROS under irradiation, Zhang et al. (76) developed a formulation for the treatment of cancer of breast. In liposomes composed of Lecithin, CHOL, DSPE-PEG2000 and PEG-NI (ethyl 6- (2-nitroimidazolyl) hexanoate coupled to PEG and chlorine e6 (Ce6) photostabilizer (6:4:0.5:0.5:0.5 molar ratio). The prodrug Tirapazamine (TPZ) and the miRNA-155 gene probe were incorporated into the liposomes. After irradiation with 670 nm laser on Ce6, the oxygen consumption for ROS generation occurs. Oxygen consumption leads to local hypoxia resulting in the reduction of prodrug TPZ to the active drug. ROS can lead cancer cells to death as well as local hypoxia, which act synergistically with TPZ chemotherapy. Finally, the miRNA-155 sonnet co-delivered with the drug could detect an oncogenic intracellular marker for diagnosis. Ce6 is added to the lipid bilayer, the incidence of the laser on it leads to destabilization of the vesicles and release of its components. This formulation was named Lip-Ce6-TPZ. The release study of TPZ was performed for the Lip-Ce6-TPZ formulation. The formulation received laser irradiation for 10 min. After 6 h, a release of 82.3% of the drug was observed. In contrast, only 29.7% of TPZ was released from the non-irradiated formulation, confirming that the laser leads to the destruction of the vesicle and therefore greater release of the drug. *In vitro*, the formation of ROS and hypoxia areas in MCF-7 cells, was 3-fold higher for treatment with Lip-Ce6-TPZ + laser compared to treatment without irradiation. *In vivo*, the relative tumor volume (RTV) in MCF-7 cell tumor bearing mice treated with Lip-Ce6-TPZ + laser was close to zero, a value seven times lower than that found for Lip-Ce6-TPZ treatment in absence of the laser (76).

In a study by Yang et al. (68) a liposome composed of PC and CHOL (4:1 weight ratio), encapsulating TPZ and the IR780 photosensitizer was developed (Lip(IR780&TPZ)). When liposomes are exposed to 808 nm irradiation the liposomal membranes rupture releasing the drug. For all concentrations of drug tested against 4T1 cells *in vitro*, irradiated liposomes lead to significantly higher cell death compared to non-irradiated liposomes. Apoptosis differed between these treatments being 36.2% and 12%, respectively. On an *in vivo* study, after 15 days of treatment mice that received the formulation followed by laser irradiation had an extremely significant reduction of tumor weight (~1g) when compared to the group that did not receive laser (~4,5g) (68).

#### Photocrosslinking, Photoisomerization, Photocleavage, and Photothermal Release

On a smaller scale, other strategies too have been reported to promote the release of liposome contents by light among them photocrosslinking, photoisomerization, photocleavage, and photothermal release (70, 77, 78). These approaches will be discussed below. The photocrosslinking is established by the polymerization of the unsaturated bonds present in the hydrophobic region of the bilayer. At the time such polymerized domains are irradiated with light at specific wavelengths, a crosslinking reaction occurs between them. From this, the lipid bilayer of the liposomes shrinks the domain where the sensitizers are present leading to a conformational change. This change in the structure leads to the formation of pores promoting a greater membrane permeability and release of the contents (79).

Yavlovich et al. (80) showed that liposomes encapsulating DXR which have the **photopolymerizable** lipid 1,2-bis (tricosa-10,12-diynoyl) sn-glycer-3-phosphocholine (DC 8.9 PC) allow superior cell death in MCF-7 breast cancer cells when exposed to light treatment (514 nm laser), compared to the same treatment without exposure to light. Formulations composed of DPPC: DC 8.9 PC: DSPE-PEG2000 (86:10:04 molar ratio) encapsulating DXR had its membranes destabilized after laser irradiation. This destabilization was accompanied by the release of DXR and cytotoxicity-enhancement cell culture, leading to 90% cell inhibition, compared to only 40% found in the absence of the laser irradiation (80).

**Figure 4:**
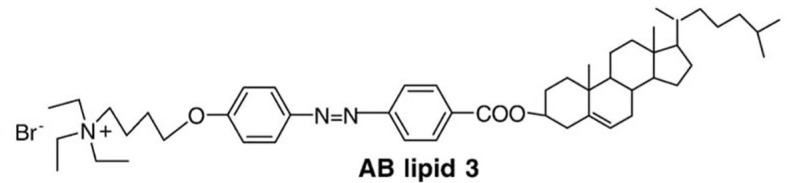
A cholesterol derivative containing portions of azobenzene Synthesized by Liu et al. (83), this derivative was called AB lipid 3.

The **photoisomerization** process is based on the conformational change (for example trans to cis) in molecules having rotating constraints, such as double bonds. This modification leads to the rupture of the lipid bilayers and expulsion of their contents (81, 82). Liu et al. (83), synthesized CHOL derivatives containing portions of azobenzenes of different polarities. These modified molecules were combined to egg PC (EPC) in the preparation of light-triggered liposomes. These liposomes were irradiated with 360 nm UV light. The conversion of the modified lipid from trans to cis was around 90% in the liposomes composed of the derivative AB lipid 3 (**Figure 4**) (EPC : AB3, 1:1 molar ratio). The release behavior of these liposomes was investigated in a calcein release study. It has been known that azobenzene derivatives in CHCl3 solution undergo trans-to-cis isomerization by UV light irradiation and cis-to-trans isomerization by visible light irradiation. Periodical UV and visible light irradiation (UV, 10 min; Visible, 15 min; both every 4 h) was carried out. at 37° C. At the end of 40 h, 55% of the calcein was released, compared to only 25% released from the liposomes not exposed to light. In this experiment, it was also verified that UV light increases the release in greater proportion compared to visible light (83).

**Figure 5:**
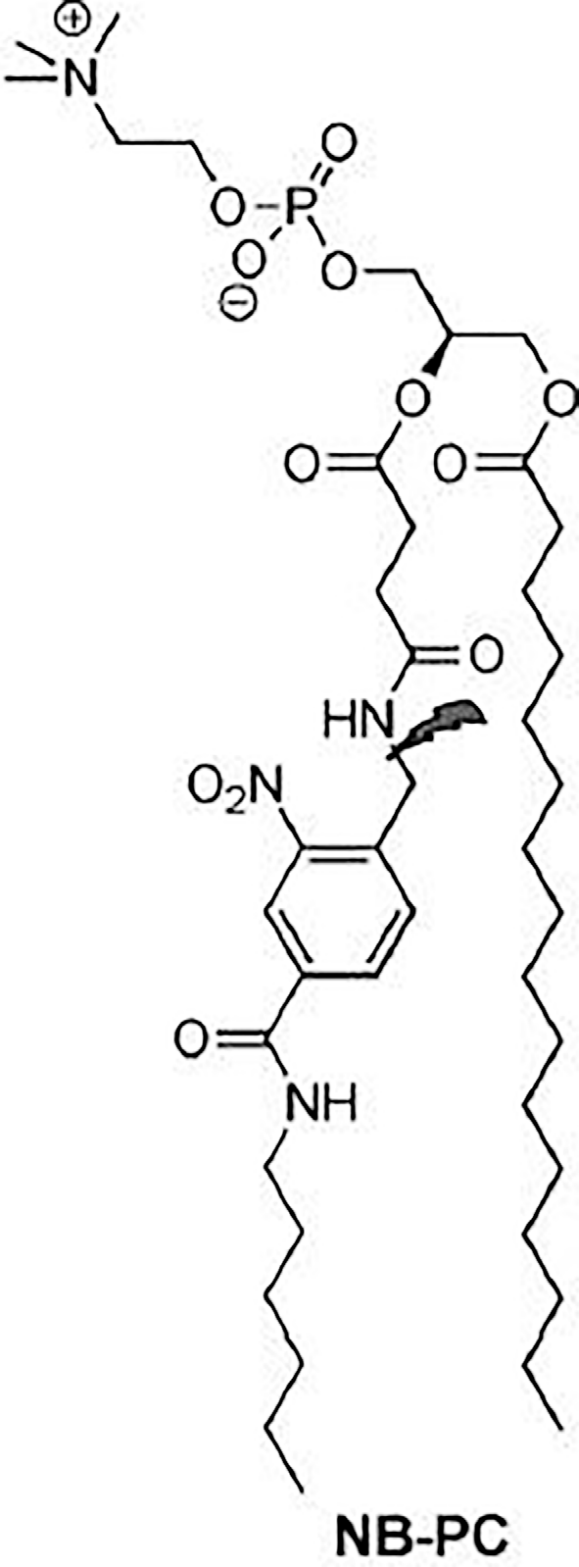
A photocleavable phospholipid derived from phosphatidylcholine Synthesized by Amichal et al. (84), this derivative was called NB-PC.

In **photocleavage**, the mechanism involves a photolabile group, for example 2-nitrobenzyl, which is inserted into the lipid bilayer. This group is cleaved after irradiation of visible/UV light. Such cleavage leads to destabilization of the vesicle membrane and release of the encapsulated contents. Amichal et al. (84) reported the synthesis of a new photocleavable phospholipid derived from phosphatidylcholine (PC), called NB-PC as shown in **Figure 5** (84).

For this, they modified the PC including a 2-nitrobenzyl group on the acyl chain at the sn-2 position. Liposomes with varying concentrations of NB-PC, phosphatidylethanolamine (PE), PEG-PE and CHOL were prepared. After irradiation of 350 nm, the release of Nile red was increased, proportionally to the increase of the modified lipid concentration. Inclusion of 10, 25, and 50% of NB-PC resulted in a gradual increase of 36, 48, and 62% in the emission of Nile red. These rates were all lower than liposomes composed exclusively of NB-PC (80%). Controls that did not receive irradiation had a minimal release. These data show that the release can be performed using a wide range of percentages of NB-PC in liposomes, and that the percentage can be used to adjust the release properties of the vesicles. It should also be noted that variations in the percentage of NB-PC had no effect on the release of non-irradiated liposomes. These liposomes ranged from 12 to 19% in, emission rate reduction, indicating that NB-PC incorporation does not destabilize the membrane peak, even for vesicles composed exclusively of the modified lipid, due to the similarity of NB-PC to natural PC (85).

Finally, the **photothermic** release consists on the conversion of light into heat in order to induce membrane permeability or rupture. The photothermal effect induces a phase transition in the bilayer, which makes it permeable increasing the release of loaded drugs. Some materials have been described for the purpose of triggering photothermal transduction, for example gold nanoparticles, carbon nanotubes and graphene-based nanosheets. After specific light irradiation, photothermal electron-rich agents can convert photon energy into vibrational energy, inducing the excitation of electrons followed by energy oscillation. Photothermal agents can transduce NIR light (wavelength between 700 and 1100 nm) to heat, triggering a local hyperthermia that can disrupt a carrier containing thermosensitive components (70, 71, 77).

Kautzka et al. (86), generated ROS in the tumor region, especially singlet oxygen using photosensitizers such as rose bengal (RB) in liposomes. For this purpose, they prepared liposomes encapsulating DXR composed of HSPC: phosphoethanolamine-N-hexanoylamine (PE-NH 2): gold nanoparticles (AuNPs) (57:5:17 molar ratio). The gold nanoparticles were added to explore the thermal property of light, due to their suitability for photothermal conversion (86). AuNPs absorb visible light and NIR and released energy as heat. The high temperatures reached by the AuNPs can induce a permeability of the lipid bilayer or its rupture, followed by the release of the charge (70). The formulation was tested *in vitro* against HCT116 human colorectal cancer cells, followed by light irradiation at 532 nm wavelength. With this study they concluded that the treatment with liposomes containing AuNPs+RB+DXR was more effective (40% cell death), than chemotherapy using the liposomes with RB+DXR (20% cell death) showing the contribution of the thermal property of light when used with photosensitizers (86).

Selection of adequate light sources determines the efficacy of light-stimulated therapies. Usually the preference is given to light with wavelengths near the infrared (NIR) range (700 nm to 2,500 nm), as they do not penetrate so deeply into the tissues (less than 1 cm) thus avoiding damage to DNA and cell proteins (87, 88). Therefore, NIR to induce drug release can be performed on diseases affecting surface tissues (88). However, the inability of light to penetrate biological tissues *in vivo* or the difficulty in matching the energy of photons from the light source is a barrier to the success of light-triggered liposomes (89). Another difficulty encountered in the pharmacotechnical development of these liposomes relates to the normally hydrophobic properties of the photosensitizers, which induces the formation of aggregates in water (87).

### Electrically-Triggered Liposomes

Electricity has several advantages as trigger mechanism for drug release compared to other types of stimuli due to the precise control of drug release as the magnitude of current and duration of electric pulse can be adjusted. Additionally, no complex instrumentation is required (90, 91).

When exposed to an external electrical field, membrane permeability of lipidic vesicles such as cells and liposomes increases because of the formation of hydrophilic pores in the lipid bilayer, on a phenomenon called electroporation (EP). Nowadays, EP is used in many fields of biology, biotechnology, and medicine (92). EP can be a permanent or transient effect. If exposure is not too long and the electric field not too strong, the pores might reseal in seconds to minutes after exposure (92–94). Reversible EP can facilitate liposome accumulation on tumor site by affecting the vascular permeability, potentially enhancing EPR effect as recently demonstrated by Srimathveeravalli et al. (2018) (95). In the same way as it is possible to get molecules into cells, it is proposed that molecules should be able to be released from liposomes (93). Irreversible EP for example, is a strategy that consists on a form of non-thermal ablation on which very high electric fields are employed to permanently compromise cell membranes (96, 97). Reversible EP is already widely used in cell culture to control diffusion of external compounds into a cell.

A great challenge behind this strategy, however, is to obtain EP conditions that will be able to trigger release from the liposomes, without damaging permanently normal cells. Different theoretical works using molecular dynamics simulations demonstrated that both the size and composition of liposomes have a great impact on EP. The amplitude needed for liposome EP strongly depends on their size, presenting a proportional inverse relationship. It has also been demonstrated that liposomes with a higher internal conductivity and lower membrane permittivity compared to other similar-sized organelles could be favorably electroporated when the pulses are few nanoseconds long. Therefore, evaluating appropriate pulse parameters and rationally designing the liposomal formulations increases the possibility of selective EP of liposomes with respect to the cell itself or its organelles (92, 94).

Yi et al. (93) designed a study to test the hypothesis that doping liposomes with amphiphilic proteins, such as nisin, could reduce the electric field required to electroporate liposomes. They prepared liposomes composed of DOPC : CHOL (10:4 weight ratio) encapsulating nisin and a fluorescent dye, CF. When exposed to 3000 V the mean release percentage of CF from the liposomes without nisin was around 12%. Liposomes containing nisin however, released approximately 14% of its content when exposed to only 200V. This significant reduction of the electric field required to release the contents of liposomes highlights the feasibility of the strategy for drug release *in vivo* (93).

### Chemically Triggered Liposomes

Chemical trigger is based on the idea of delivering an exogenous chemical to trigger a previous administered nanosystem. There are some advantages of this strategy in detriment of other external triggering methods: 1) chemical triggers are able to reach the nanosystem on any part of the body, dismissing the need of knowing the exact location of the tumor; 2) they are able to efficiently reach deep tissues and 3) no technological and expensive equipment are necessary. Many chemicals are known to disrupt the membrane bilayer; however, this strategy is still poor developed. That is probably due to the great challenge of finding a liposomal composition that is selectively destroyed by an exogenous chemical trigger which on the other hand has little affinity to the host cell membranes (98, 99).

Xiong et al. (12) demonstrated that chloroquine (CQ) triggers the release of daunorubicin (DAU) from liposomes composed of HSPC : CHOL:mPEG2000-DSPE:folate–PEG–CHEMS (55:40:4.5:0.5, molar ratio). As CQ was added to the formulation, it quickly loaded into liposomes expelling the DAU, possibly through a mechanism involving intraliposomal pH rising. An enhancement in cytotoxicity against L1210JF (murine lymphocytic leukemia) cell line was observed when CQ was added to DAU liposomes. The observed IC50 were 20.0 ± 1.8 µM without CQ and 11.2 ± 1.2 µM in the presence of 10 µM of CQ, a concentration supposed to be non-toxic. However, the *in vitro* gains did not translate to *in vivo* using a DBA/2 mice carrying L1210JF tumors (12).

**Figure 6:**
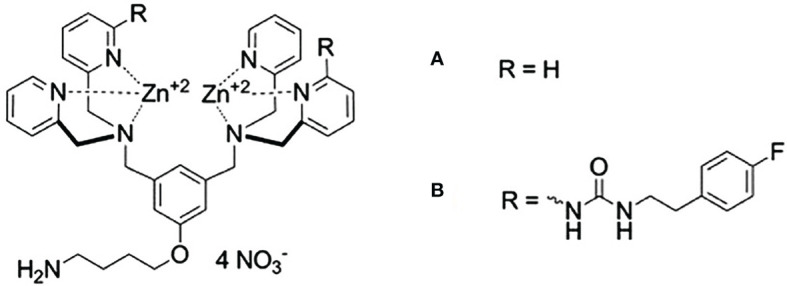
Zinc(II)-dipicolylamine (ZnDPA) complexes of first **(A)** and second generation **(B)**. Designed by Plaunt et al. (98), these complexes were used as triggering agents for anionic liposomes.

Plaunt et al. (98) reported that a zinc(II)-dipicolylamine (ZnDPA) complex, shown in **Figure 6** can act as a chemical trigger by associating selectively with anionic bilayer membranes (such as from liposomes containing phosphatidylserine, PS), inducing the leakage of water-soluble contents. Healthy mammalian cells present zwitterionic membranes, which are not targeted, insuring the selectivity of the trigger agent. They prepared liposomes composed of DPPC : CHOL : POPS (67:28:5 molar ratio) and showed that, in a CF leakage assay, it released 55% of its content in 120 s, when exposed to 10 µM of the ZnDPA complex. A negligible CF leakage (3%) was observed for zwitterionic liposomes mimicking healthy cells, composed DPPC: CHOL (67:28 molar ratio), under the same conditions. Knowing that PS-rich liposomes need an extensive steric protection to prevent its capture by the MPS, they also tested a formulation containing PEG. This formulation, composed of DPPC : CHOL : POPS : DPPE-PEG2000 (67:28:5:8 molar ratio), released 84% of its CF content in 120 s when exposed to the ZnDPA complex. This higher trigger for a sterically protected liposome could be explained by the fact that the DPPE-PEG2000 is also anionic, and could associate with the ZnDPA complex. Liposomes exposed to the ZnDPA complex showed no significant change in the hydrodynamic diameter, which was evidence against liposome fusion and against a lysis process. They proposed that association of cationic ZnDPA complex with the anionic POPS head group induces domain formation and perhaps phase separation. This creates line tension and mismatched membrane thickness at the domain interfaces, promoting drug leakage (98).

This initial release studies were performed in TES (N-Tris(Hydroxymethyl)Methyl-2-aminoethane sulfonic acid) buffer. When release was later evaluated by Plaunt et al. (99) in phosphate buffered saline, which is closer to a physiologically relevant media, the DPPE-PEG2000 containing formulation released only 34% of its content. That motivated the search for a next-generation chemical trigger that operates more effectively than the old one. They obtained the modified ZnDPA complex, shown in **Figure 6B**, and showed that DPPC : CHOL : POPS : DPPE-PEG2000 (67:28:5:8 molar ratio), released 71% of its CF content in 120 s when exposed to the modified ZnDPA complex in PBS. Formulation was used to encapsulate 5-Aminolevulinic acid (5-ALA) and evaluated for its release. In the absence of chemical trigger the liposomes remained intact, releasing <10% of the encapsulated 5-ALA and upon exposure to the chemical trigger, 80% release of the encapsulated 5-ALA was observed after 24 h (99).

On **Table 1**, we summarize the most important aspects of important liposomal formulations triggered by different exogenous stimuli developed to date.

**Table 1:** Summary of important liposomal formulations triggered by different exogenous stimuli.

Trigger stimulus	Lipid composition	Encapsulated agent(s)	Encapsulated percentage	Mean diameter	Reference
**Thermo**	DPPC : DSPC (7:3 weight ratio)	Methotrexate	N.A.	N.A.	(25)
**Thermo**	DPPC : HSPC : CHOL : DPPE-PEG (50:25:15:3 molar ratio)	DXR	N.A.	127 nm	(26)
**Thermo**	DPPC : MPPC : DSPE-PEG2000 (90:10:4 molar ratio)	DXR	N.A.	~ 140 nm	(11)
**Thermo**	DPPC: MSPC: DSPE-PEG2000 (86:10:4 molar ratio)	DXR	N.A.	N.A.	(27)
**Thermo**	DPPC/DSPC/DPPG2 (50:20:30 molar ratio)	–	N.A.	175 nm	(35)
**Thermo**	DPPC : Brij78 (96:4 molar ratio)	DXR	N.A.	N.A.	(36)
**Thermo**	DPPC: 1-StePc: DSPE-PEG2000 (86:10:4 weight ratio)	MATT	56%	100 nm	(34)
**Thermo**	HSPC : CHOL : DSPE-PEG-NHS (75: 50: 3 molar ratio) with elastin-like polypeptides modified surface	DXR	N.A.	151 nm	(38)
**Magnetic** **(HF)**	DPPC : CHOL (5:1 weight ratio)	dextran-coated iron oxide MN (3–5 nm); carboxylfluorescein (CF)	N.A.	150–230 nm	(52)
**Magnetic** **(HF)**	DPPC : CHOL : DSPE-PEG2000:DSPE-PEG-2000-Folate (80:20:4.5:0.5 molar ratio)	Iron oxide MN (10 nm); DXR	about 24% and 85%, respectively	360 nm	(53)
**Magnetic** **(HF)**	DSPC: MPEG-2000-DSPE (10:0.5 molar ratio)	palmityl-nitroDOPA-stabilized iron oxide MN (5–10 nM); calcein.	N.A.	N.A.	(54)
**Magnetic** **(HF)**	HSPC/DSPE/CHOL (12.5:1:8.25 molar ratio)	citric acid-coated iron oxide MN (10 nm); DXR	15.5% for DXR	130 nm	(55)
**Magnetic** **(HF)**	DPPC : CHOL : MPEG-2000-DSPE (80:20:5 molar ratio)	Oleic acid coated iron oxide MN (10 nM); curcumin	76.15% for curcumin	120 nm	(57)
**Magnetic (LF)**	PC : CHOL:amphiphilic carboxymethyl dextran (CMD) (55:40:0.5 molar ratio)	citric acid-coated Iron oxide MN (3–5nm); DXR	96.9% for DXR	220 nm	(56)
**Ultrasound**	DPPC: MPPC: DSPE-PEG2000: DSPE-PEG-2000-iRGD (86: 10: 2: 2 molar ratio)	DXR	>95%	94.2 ± 2.0 nm	(62)
**Ultrasound**	DPPC: MSPC: DSPE-mPEG2000 (85.3: 9.7: 5.0 molar ratio)	DXR + PFP gas	≅ 67% DXR	171.6 ± 0.5 nm	(63)
**Ultrasound**	soy lecithin and CHOL/CHOL-TPP (0.7/0.3 weight ratio)	hematoporphyrin monomethyl ether	74,6%	110 nm	(64)
**Ultrasound**	DMPA: DPPC: CHOL (1:4:5 molar ratio) avidin/HVJ	perfluoropentane	N.A.	N.A.	(65)
**Light**	DSPC: DOPC: CHOL: Porphyrin-phospholipid (54.7: 5: 40: 0.3 molar ratio)	DXR	≅ 95%	~ 120 nm	(72)
**Light**	DSPC : CHOL : DSPE-PEG2000 : PoP (53:40:5:2 molar ratio)	DXR	≅ 95%	~ 100 nm	(73)
**Light**	DSPC: DOPE: CHOL: DSPE-PEG2000 (85: 10: 5: 5 molar ratio)	talaporfin sodium (TPS)+ gemcitabine (GEM)	TPS 11.6 ± 3.0% and GEM 2.3 ± 0.5%, respectively	115.8 ± 3.8 nm	(74)
**Light**	1- (1z-octadecenyl) -2-oleoyl-sn-glycero-3-phosphocholine (PLsPC): CHOL : DSPE-PEG-2000: soya bean lecithin (S100) [S100:CHOL (5:1); S100:PLsPC (4:1 or 2:1)] (molar ratio)	DXR	N.A.	128.1 ± 1.8 nm	(75)
**Light**	Lecithin: CHOL: DSPE-PEG2000: PEG-NI (ethyl 6- (2-nitroimidazolyl) hexanoate coupled to polyethylene glycol): chlorine e6 (6: 4: 0.5: 0.5: 0.5 molar ratio)	Tirapazamin	N.A.	162 ± 4 nm	(76)
**Light**	PC : CHOL (4 :1 weight ratio)	Tirapazamin	51,84%	130 nm	(68)
**Light**	DPPC: DC 8.9 PC: DSPE-PEG2000 (86:10:04 molar ratio)	DXR	1335 ng per nmol Pi	159.9 ± 6 nm (25°C)	(80)
**Light**	EPC: AB3 (1: 1 molar ratio)	Calcein	N.A	N.A	(83)
**Light**	NB-PC : PE: PEG-PE : CHOL (various concentrations)	Nile red	N.A	N.A	(85)
**Light**	HSPC: phosphoethanolamine-N-hexanoylamine (PE-NH 2): gold nanoparticles (AuNPs) (57: 5: 17 molar ratio)	DXR + rose Bengal (RB)	23.4% e 88% respectively. N° of gold nanoparticles in each liposme ~ 109.	124.6 ± 2.3 nm	(86)
**Electrically**	DOPC : CHOL 10mg:4mg	Carboxyfluorescein and nisin 2.5 mg/L	N.A.	N.A.	(93)
**Chemical** **(**chloroquine)	HSPC : CHOL:mPEG2000-DSPE:folate–PEG–CHEMS (55:40:4.5:0.5, molar ratio)	daunorubicin	95%	N.A.	(12)
**Chemical** **(**ZnDPA complex)	DPPC : CHOL : POPS : DPPE-PEG-2000 (67:28: 5:8 molar ratio)	5-ALA	2%	200 nm	(98, 99)

## Triggered Drug Release by Endogenous Stimuli

### PH-Triggered Systems

The pH of normal tissue and blood under physiologic conditions is around 7.4. However, the extracellular pH of malignant tumors is lower than normal tissue, with average pH values of 6.8. Intracellular compartments such as endosomes and lysosomes are the most acidic with pH values between 4.5–6.5. pH-sensitive liposomes have been developed as anticancer agent’s delivery systems mainly due to their ability to fuse with the endosomal membrane, releasing their content into the cytoplasm. This allows the accumulation of anticancer drugs in tumors as a specific release of the drug controlled by the tumor environment occurs. Besides that, the intracellular delivery of anticancer agents by pH-sensitive liposomes presents an efficient mean to overcome the multidrug resistance, one of the main causes of tumor recurrence (100–103).

**Figure 7:**
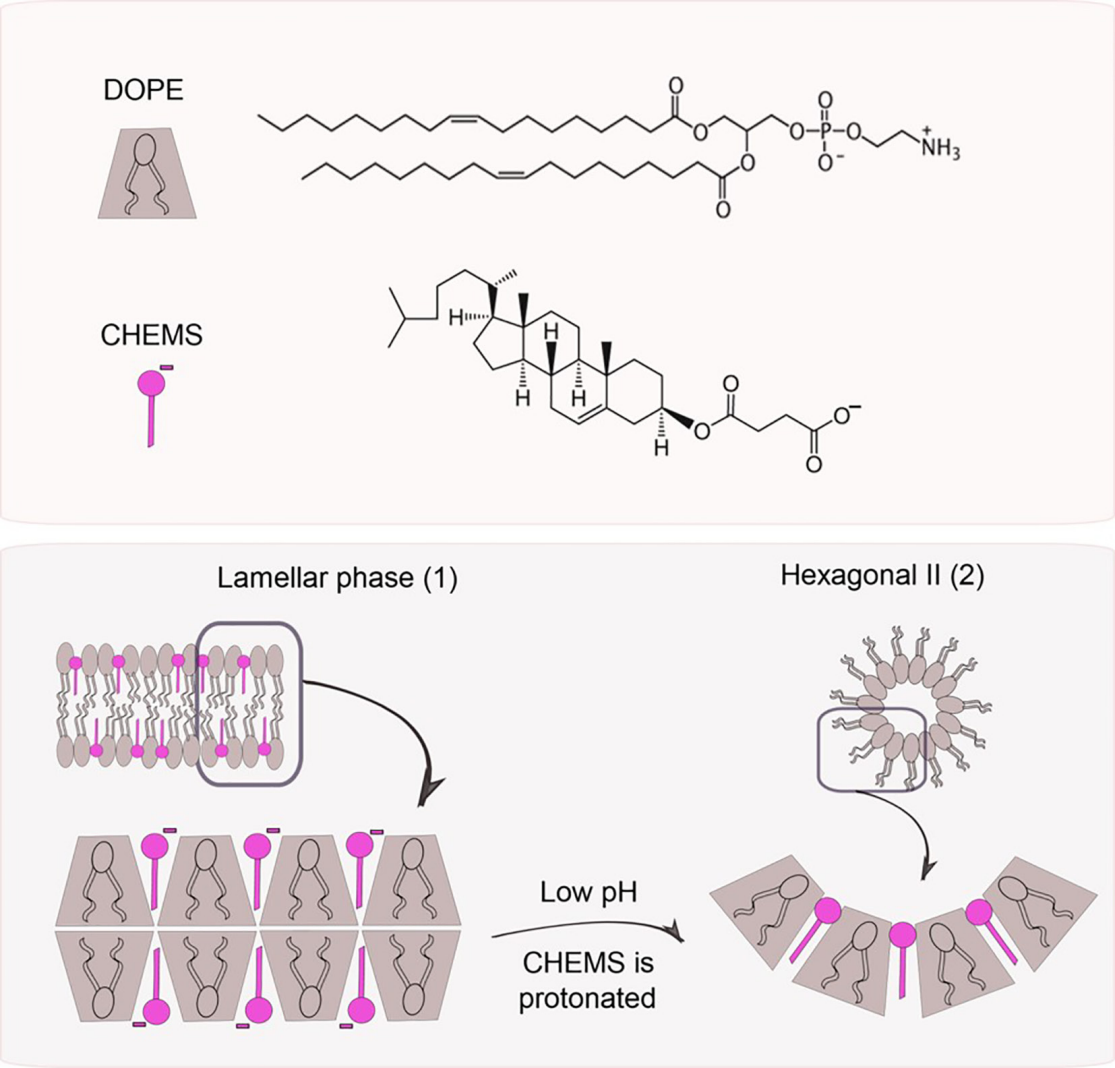
pH triggers phase changes in liposomes composed of dioleoylphosphatidylethanol-amine (DOPE) and cholesteryl hemisuccinate (CHEMS). When alone, DOPE molecules organize themselves in an inverted hexagonal phase due to their conic geometry. When combined with CHEMS at physiological pH, it is possible to obtain a bilayer (lamellar phase, 1). Once exposed to acidic pH, the protonation of carboxylate groups takes place leading to liposome destabilization (hexagonal phase II, 2), followed by the release of the encapsulated agents.

The polymorphic lipid PE or its derivatives, such as dioleoylphosphatidylethanolamine (DOPE), are the most common used molecules to obtain pH-sensitive liposomes (101). At neutral or physiological pH, these molecules are not able to organize themselves in bilayers, inducing the inverted hexagonal phase (H_II_) organization, due to the small volume of the polar head compared to the hydrocarbon chain, favoring strong intermolecular interactions between amine and phosphate groups (101, 104). The insertion of an amphiphilic acid, such as cholesteryl hemisuccinate (CHEMS), at neutral pH, causes an electrostatic repulsion between phosphate and carboxylate groups, favoring the formation of lamellar phases (105). When exposed to the acid environment, such as endosomes, the carboxyl group of CHEMS is protonated causing an electrostatic repulsion which results in a transition from lamellar phase to H_II_ and consequent release of the encapsulated drug, as represented in **Figure 7** (101, 105).

pH-sensitive liposomes incorporating estrone derivative conjugated to DSPE-PEG (ES-PEG-DSPE), were developed by Paliwal et al. (106) to targetly deliver DXR to estrogen receptors (ER)-bearing tumor cells. This liposome was composed of DOPE : HSPC : CHEMS : CHOL : ES-PEG-DSPE (ES-pH-SL). As control, a non-pH sensitive liposomes (ES-SL) was prepared with HSPC : CHOL : ES-PEG-DSPE. ES-pH-SL and ES-SL were prepared at various molar ratios with ES-PEG-DSPE at 5 mol % to phospholipids. The DXR encapsulation efficiency was similar for liposomes with different compositions (approximately 90%). To evaluate pH-sensibility, the release profile of DXR from liposomes was investigated at different pH values. The DXR release from ES-pH-SL within 2 h at pH 5.5 was 90%, compared to only 40% after 24 h at pH 7.4. However, the release of DXR from ES-SL was approximately same, at both pH, about 30% after 24 h. The ES-pH-SL at pH 5.5 exhibited a 6-fold increase in mean diameter, indicating vesicle aggregation and/or membrane fusion, but ES-SL did not show any difference in vesicle size at the same pH. *In vivo* biodistribution and antitumor efficacy were evaluated in female Balb/C mice with 7,12-dimethylbenz[a]anthracene (DMBA) induced breast tumor. After DXR administration at a dose of 5 mg/kg, the DXR concentration in tumor tissue was about 1.4-fold higher for ES-pH-SL compared to ES-SL. In addition, the DXR concentration of the ES-pH-SL in the heart was significantly lower than ES-SL or free DXR, an important result facing DXR cardiotoxicity. The tumor volume in the control group increased rapidly over 30 days (increase of 40%). The treatment of mice with ES-pH-SL significantly reduced the tumor volume (reduction of 80%) compared to those animals treated with ES-SL or free DXR (reductions of 60% and 20%, respectively), in relation to the tumor volume of the control group at the start of drug treatment (day 0) (106).

Silva et al. (107) prepared long-circulating pH-sensitive liposomes encapsulating DXR (SpHL-DXR) composed of DOPE : CHEMS : DSPE-PEG2000 (5.7:3.8:0.5 molar ratio). As control, non-pH-sensitive liposomes were prepared, composed of HSPC : CHOL : DSPE-PEG2000 (5.7:3.8:0.5 molar ratio) (nSpHL-DXR). In order to confirm the pH sensibility of the formulation and track liposome´s fate *in vivo*, blank liposomes were incubated with the radiolabeled complex [^99m^Tc]-DXR as an imaging probe. The pH sensitivity for SpHL-[^99m^Tc]-DXR was confirmed in a study of [^99m^Tc]-DXR leakage at different pH, showing a higher release in acidic conditions (51.3%, pH=6.0) compared to neutral condition (13.5%, pH=7.4). This enhanced release at pH=6.0 was significantly higher compared to the release from nSpHL-[^99m^Tc]-DXR in the same pH (30.8%). The biodistribution profile of SpHL-[^99m^Tc]-DXR and nSpHL-[^99m^Tc]-DXR were evaluated after intravenous injection in Balb/C female mice bearing 4T1 breast tumor. A higher uptake of SpHL-[^99m^Tc]-DXR and nSpHL-[^99m^Tc]-DXR by the tumor site was observed when compared to the contralateral muscle. Additionally, the tumor-to-muscle ratio observed for SpHL-[^99m^Tc]-DXR was significantly higher (approximately 2-fold) than the observed for nSpHL-[^99m^Tc]-DXR at 4 and 24 h, suggesting that the pH sensitivity contributed to higher uptakes of [^99m^Tc]-DXR by the tumor (107).

In order to improve liposomal endocytosis, Silva et al. (108) modified the surface of the SpHL-DXR with folic acid, aiming on the overexpressed folate receptors (FR) on the surface of breast cancer cells. These folate-coated, long-circulating and DXR-loaded pH-sensitive liposomes (SpHL-DXR-Fol) were composed of DOPE, CHEMS, DSPE-PEG2000, and DSPE-PEG2000-Fol (5.8:3.7:0.45:0.05 molar ratio). The non-folate-coated SpHL-DXR were used as control. The pH-sensitivity of SpHL-DXR-Fol was determined by dialysis in HEPES-saline buffer. After 24 h incubation at pH 5.0, DXR leakage of 53.6% was observed for the SpHL-DXR-Fol, compared to only 21.5% leakage at pH 7.4. The antitumor activity was evaluated *in vivo* using a 4T1 breast cancer model. The results showed that SpHL-DXR-Fol treatment was the most effective, presenting a higher inhibition ratio (IR) of the RTV compared to SpHL-DXR and free DXR (68%, 56% and 37%, respectively) (108).

Another way to develop pH-sensitive liposomes is by insertion of a pH-responsive peptide that change their conformation in acid ambient, causing destabilization of the liposomes. Also, responsive polymers can be used to produce these pH sensitive systems (101, 109). Some polymers used in pH-sensitive liposomes destabilize the phospholipid bilayer, while others cause the fusion of the liposome with endosome/lysosome membranes (103).

Chiang et al. (110) developed liposomes composed of DPPC and a pH responsive polymer methoxy-poly(ethyleneglycol)-b-poly(N-2-hydroxypropyl methacrylamide-co-histidine)-cholesterol (mPEG-P(HPMA-g-His)-CHOL) linked by biotin-PEG-biotin (biotin2-PEG) (34:1:12,5 molar ratio). These liposomes were used to encapsulate DXR, and evaluated in colon rectal cancer. When exposed to acidic environment the imidazole ring of histidine in the polymer is protonated generating a repulsive force that destabilizes the liposome lipidic bilayer releasing the DXR. When tested *in vitro*, the DXR release from sensitive liposomes was 90% in pH 5.0 and 30% in pH 7.4. On the other hand, the conventional liposomes, composed by DPPC without polymer, released only 30% of DXR content in both pH. The IC50 values of polymer-biotin-sensitive liposome for HCT116 colon rectal cancer cells at pH 7.4 was 19.3 µg/ml, which was similar to free DXR and non-sensitive liposome. When evaluated for the antitumor efficacy in mice bearing HCT116 the inhibition of tumor growth in mice receiving pH sensitive liposomes was near to 90%, while that for non-responsive formulation was only near to 70% (110).

In a study by Zhao et al. (111) DXR was encapsulated in liposomes composed of DOPE: CHEMS: DSPE-PEG: DSPE-PEG-H_7_K(R_2_)_2_ (65:30:4:1 molar ratio). The tumor-specific pH-responsive peptide H_7_K(R_2_)_2_ (the sequence is RRK(HHHHHHH)RR) contains the cell-penetrating oligoarginine (R_2_)_2_ and the pH trigger oligohistidine H_7_. As control liposomes DXR-PSL and DXR-SSL were prepared with DOPE : CHEMS : DSPE-PEG and EPC : CHOL : DSPE-PEG (65:30:5 molar ratio), respectively. The release of DXR from DXR-SSL, DXR-PSL and DXR-PSL- H_7_K(R_2_)_2_ were evaluated in pH 5.5, 6.0, 6.5, and 7.4 for 24 h. To SSL after 24 h the content of DXR released was near to 20% in the four different pH evaluated. The release profiles of DXR-PSL and DXR-PSL- H_7_K(R_2_)_2_ were similar, near to 20% in pH 7.4 and near to 80% in pH 5.5, 6.0, and 6.5. The coumarin-6 uptake by C6 and U87 cells from coumarin-6-SSL, coumarin-6-PSL and coumarin-6-PSL- H_7_K(R_2_)_2_ were analyzed by flow cytometry. The fluorescence intensity of coumarin-6 in pH 7.4 were almost identical to the three formulations. However, in pH 6.8 the fluorescence intensity of coumarin-6 from coumarin-6-PSL and coumarin-6-PSL- H_7_K(R_2_)_2_ were higher than coumarin-6-SSL about 1.2 and 1.7-fold, respectively. The C6 cell line was used to investigate the *in vitro* cytotoxicity of liposomes. At pH 7.4, the IC_50_ values determined for DXR-SSL (14.9 ± 2.4 µg/ml), DXR-PSL (12.4 ± 1.7 µg/ml) and DXR- PSL- H_7_K(R_2_)_2_ (12.6 ± 0.7 µg/ml) were similar. At pH 6.8, the IC_50_ values determined for DXR-SSL (12.2 ± 0.3 µg/ml), DXR-PSL (3.7 ± 0.5 µg/ml) and DXR- PSL- H_7_K(R_2_)_2_ (3.2 ± 0.4 µg/ml), unlike DXR-SSL group, were significantly reduced compared with that at pH 7.4. The antitumor efficacy was evaluated in human C6 xenograft model and after 21 days the mean tumor sizes determined for DXR-SSL, DXR-PSL and DXR-PSL-H_7_K(R_2_)_2_ groups were 4369 ± 793, 1989 ± 205 and 1148 ± 285 mm^3^, respectively, confirming that the pH-sensibility by polymorphic lipid and peptide can be a good strategy to produce an efficient responsive system to anticancer drug delivery (111).

### GSH-Triggered Liposomes

**Figure 8:**
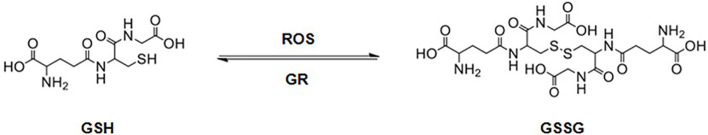
Glutathione (GSH) is oxidized to glutathione disulfide (GSSG) in the presence of reactive oxygen species (ROS). GSSG is reduced back to GSH by glutathione reductase (GR).

Aerobic life demands oxygen, and the respiration process leads to the formation of ROS that leads to oxidative stress. Glutathione (L-γ -glutamyl-L-cysteinylglycine, GSH), is one of the most important cellular antioxidant systems (112). GSH protects the biological system from oxidizing factors, such as ROS, by terminating them. In this process, GSH itself is oxidized to glutathione disulfide (GSSG), which is reduced back to GSH by glutathione reductase (GR), as shown in **Figure 8** (113).

GSH concentrations vary both from extracellular to intracellular environment and from normal to tumor cells. GSH concentrations in blood and extracellular matrix have been reported to be up to 1000-fold lower than that observed in the intracellular environment. In tumor cells, GSH levels have been reported to be up to 100-fold higher than that of normal cells. This high redox potential difference allows for the design of nanosystems that are selectively triggered in the tumor tissue (114).

**Figure 9:**
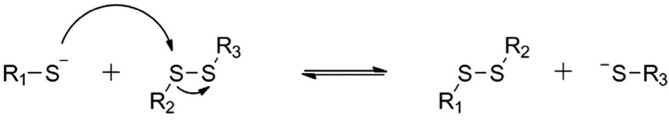
The thiol disulfide exchange reaction.

Different nanocarriers have been designed upon this concept such as micelles (115), nanoparticles based on hydroxyethyl starch (116), gold-nanoparticle (117) and liposomes (118–120). Most of the materials used to obtain these nanosystems contain characteristic disulfide (S-S) bonds. These bonds are highly stable when exposed to low levels of GSH, like that on extracellular environments, but tend to be rapidly cleaved in the intracellular highly reducing environments, by GSH mediated thiol-disulfide exchange reaction. **Figure 9** represents this reaction, which starts with the nucleofilic attack of a disulfide bond by a deprotonated thiol resulting on the S-S bond cleavage producing a new disulfide and a new thiol (121).

Fu et al. (118) prepared redox-sensitive liposomes modified with TAT, a cell-penetrating peptide. The formulation was composed of CHOL, SPC, DSPE-S-S-PEG5000, and DSPE-PEG2000-TAT (10:69.5:10:0.5 molar ratio) and used to encapsulate PTX. When evaluated against murine melanoma B16F1 tumor xenograft model, in the end of 14 days the RTV for animals treated with the redox-sensitive TAT modified liposomes (~250%) was significantly lower compared to animals treated with non-redox sensitive and non TAT modified liposomes (CHOL : SPC:DSPE-PEG2000, 10:75:5 molar ratio; RTV ~700%) and compared to non-redox sensitive TAT modified liposomes (CHOL : SPC:DSPE-PEG5000: DSPE-PEG2000-TAT, 10:69.5:10:0.5 molar ratio; RTV ~400%). These results showed that both the S-S binder and TAT modification had an important role in enhancing the antitumor efficacy (118).

Chi et al. (122) prepared redox-sensitive liposomes composed of SPC, CHOL, DOTAP, DOPE, and PEG2000 conjugated with CHOL through a bio-reducible disulfide linker (CHOL-SS-mPEG) at a 4:1:2:2:1 molar ratio. Non redox-sensitive liposomes were prepared using CHOL-mPEG instead of CHOL-SS-mPEG. These liposomes were coated with hyaluronic acid, a ligand to CD44, and used to encapsulate DXR. An *in vitro* burst release was observed for this formulation in the presence of 10 mM GSH with >60% of DXR released in the first 4 h. In contrast, the non-redox sensitive liposomes released only approximately 30% of DXR after 72 h, independent of the GSH concentration. When tested for antitumor activity against xenograft osteosarcoma (MG63) mouse model, the redox-sensitive liposomes showed the best efficacy (tumor volume ~0.5cm³) compared to non-redox-sensitive liposomes (tumor volume ~1.25cm³) and free DXR (tumor volume ~1.75cm³) in the end of 24 experimental days (122).

Chen et al. (120), prepared redox-sensitive oligopeptide liposomes composed of the redox-sensitive cationic lipid (LHSSG2C14), natural soybean phosphatidylcholine (SPC) and CHOL (5:5:1 weight ratio) encapsulating PTX and anti-survinin siRNA. The formulation was evaluated for PTX and siRNA release *in vitro* in GSH concentrations of 10 μM, which simulated the extracellular environment, and 10 mM, which simulates the intracellular environment. When exposed to 10 μM of GSH, less than 30% of PTX was released in 36 h. When the GSH concentration was 10 mM, the accumulated release of PTX was more than 80% in 36 h. In comparison, a non-redox sensitive formulation (prepared with LHG2C14 instead of LHSSG2C14) released less than 25% of PTX in 36h when GSH concentration was 10 mM. The bands of siRNA in agarose electrophoresis experiment could be detected after 2 h of incubation with 10 mM GSH but were not detected after incubation with 10 μM of GSH even after 8 experimental h. These results implied a relative stability of the formulation under the physiological conditions with targeted release in the presence of high GSH concentrations. When evaluated for its cytotoxicity, the redox-sensitive liposomes presented an IC50 value of 0.35 μg/ml against 4T1 breast cancer cells, while the non-redox sensitive liposomes and Taxol^®^ presented IC50 values of 0.82 μg/ml and 0.85 μg/ml, respectively (120).

### Enzyme-Triggered Liposomes

Enzymes are proteins with extreme biorecognition ability and catalytic function in chemical reactions. They can be used as diagnostic markers for pathologies or target-therapeutics, since some diseases present differences in their expression compared to healthy tissues (123, 124). The over expression of proteases and phospholipases in tumor tissue, when compared to the normal tissue, has been explored to develop enzyme-triggered liposomes (125, 126). Enzyme-responsive liposomes release their content in response to pathologically increased enzyme levels at the target site (127).

### Protease Triggered Liposomes

Proteases are responsible for breaking peptide bonds between the amino acids of proteins. Metalloproteases (MMP) are proteases that attack collagen, elastin, fibronectin and proteoglycan, which causes the degradation of extracellular matrices. MMP-2 e MMP-9 are overexpressed in almost all cancers, being an attractive target to develop enzyme-triggered delivery systems (126).

Sarkar et al. (128) explored the MMP-9 to trigger liposomes composed of DSPC and a lipopeptide (LP1; [CH_3_(CH_2_)_16_COHNGPQGIAGQR(GPO)_4_GG-COOH]). The MMP-9 recognizes and unwinds the triple helical structure of LP1 on the liposomal surface, resulting in their destabilization and release of the encapsulated material. For release studies, liposomes composed of DSPC : LP1 (90:10 molar ratio) or 100% DSPC (control) were loaded with CF dye. When exposed to MMP-9 for 5 h, the responsive liposomes released around 55% of CF, while no CF release was observed for control liposomes. In contrast, without MMP-9, the responsive liposomes released only 10% of the dye, confirming the triggering potential of the enzyme (128).

In a study by Kulkarni et al. (129), gemcitabine was encapsulated in liposomes composed of 1-palmitoyl-2-oleoyl-sn-glycero-3-phosphocholine (POPC): LP1: PEGylated 1-palmitoyl-2-oleoyl-sn-glycero-3-phosphoethanolamine (POPE-S-S-PEG5000): CHEMS: lissamine rhodamine lipid (59:30:5:5:1 molar ratio). These liposomes were designed to be sequentially GSH and enzyme (MMP-9) triggered. The disulfide bonds of POPE-S-S-PEG5000 are cleaved by GSH mediated reaction, resulting in the exposure of LP1 that is hydrolyzed by MMP-9. Free and liposome-encapsulated gemcitabine showed similar cytotoxicity for the PANC-1 cells (viability around 30-35%) and MIAPaCa-2 cells (viability around 45-50%). The higher cytotoxicity in PANC-1 cells was suggested to be due to the higher concentration of MMP-9 measured on the conditioned media of PANC-1 (126 ± 23 pg/ml) compared to that of MIAPaCa-2 cells (8 ± 4 pg/ml). The antitumor efficacy was evaluated in nude-Foxn1 mice with PANC-1 xenograft tumor. Animals treated with gemcitabine (10 mg/kg/week) encapsulated in MMP-9 responsive liposomes presented lower increase in tumor volume (increase of 125%) when compared to gemcitabine encapsulated in liposomes without LP1 (increase of 175%) in the end of 4 weeks (129).

### Phospholipase Triggered Liposomes

Phospholipase A2 (PLA2) degrades phospholipids and is over expressed in a variety of cancer types, namely prostate, lung, breast and pancreatic (126, 127). This catalytic activity is enhanced when phospholipids are organized, such as in liposomes, compared to lipid monomers; and is dependent on the lipid composition and membrane charge. The negative charge of phospholipids has also an important role in activity of the PLA2 (130). PLA2 acts at the lipid−water interface. Initially, interacts with the membrane by its interfacial binding surface and later the lipid is hydrolyzed in the active site of the enzyme (127).

Mock et al. (133) prepared two different PLA2 responsive liposomes composed of DSPC : DSPE-PEG : CHOL : DSPG (SPRL-G) (8:1:5:1 molar ratio) and of DSPC : DSPE-PEG : CHOL : DSPE (SPRL-E) (8:1:5:1 molar ratio) and a control liposome, without negative charge, composed of DSPC : DSPE-PEG : CHOL (9:1:5 molar ratio). CF was added to the formulations on the lipidic film hydration and DXR was encapsulated by remote loading. Release studies showed that CF released from the different liposomal formulations was negligible in the end of 108 h when PLA2 was absent. In contrast, in the presence of PLA2 there was an increased CF release from control liposomes (11%) SPRL-E (11%) and SPRL-G (14%) in the end of 108 h. A significant CF release from SPRL-E and SPRL-G started at 24–36 h, but for control liposomes it started only after 48 h. The antitumor efficacy of SPRL-E and the control liposomes was evaluated in human PC-3 xenograft model, that received the treatments containing 5mg/Kg of DXR on a weekly basis for four weeks. After 35 days, the tumor volume in the SPRL-E and control groups were 300mm^3^ and 500mm^3^, respectively, suggesting that SPRL-E are more effective for tumor growth inhibition (131).

### Hypoxia-Triggered Liposomes

The rapid growth of tumor cells demands a large amount of oxygen. As the tumor vasculature is irregular and abnormal, the oxygen supply is deficient, thus resulting in hypoxic regions. The hypoxia is a pathological phenomenon that consists in an important hallmark of tumor microenvironment. The oxygen partial pressure on some tumor tissues can be as low as 5–10 mm Hg, compared to the healthy tissue where it is near 30–50 mm Hg (132, 133). Due to this significant difference in the amount of oxygen, hypoxia is a promising target for cancer therapy (124). It is reported that the pathological phenomenon of hypoxia causes an increase in reductive stress, resulting in overexpression of nitroreductase, azoreductase, and quinone reductase (134).That in mind, pro-drugs that are activated to form the cytotoxic agent in hypoxia regions were designed (132), as well as hypoxia-triggered drug delivery systems to release drugs to hypoxic sites (133).

Liu et al. (135) obtained a new lipid called MDH. For that, first the hypoxic radiosensitizer 2-Methyl-5-nitroimidazole-1-ethanol (metronidazole) was conjugated to hexadecanedioic acid (HA) with a hydrolysable ester bond, in order to form (16-(2-(2-methyl-5-nitro-1H-imidazol-1-yl) ethoxy)-16-oxohexadecanoic acid (MHA). The MHA was then coupled with 3-dimethylaminopropane-1, 2-diol (DA) to form ester linked MDH. The tertiary amine group on the MDH becomes protonated when exposed to the acidic ambient of tumor, enhancing the cellular uptake of liposomes. The nitro group on the MDH can be converted to a hydrophilic amino group by nitroreductases in hypoxic conditions, leading to the destabilization of the liposomes and the release of its content. The MDH lipid was used for obtaining hypoxia-responsive liposomes composed of DSPE-PEG2000: MDH: CHOL (1:6:3 molar ratio) encapsulating DXR. These liposomes were evaluated for DXR release and in 5 h under hypoxic conditions 65.78% of the drug was released, versus approximately 25% in normoxic conditions. As a control, non-hypoxia-responsive liposomes composed of *1,2-Bis(palmitoyloxy)-3-(dimethylamino) propane (*PD): DSPE-PEG2000: CHOL (1:6:3 molar ratio) encapsulating DXR were prepared. These liposomes have the tertiary amine as well as the MDH-liposomes, but it does not present the hypoxia-responsive nitro group.

The hypoxic-responsive DXR release from hypoxia-responsive liposomes was investigated. Under hypoxic conditions, the liposomes released 65.8% of its DXR content within 5h while no significant DXR release was observed under normoxic conditions. The rapid DXR release of the hypoxia-responsive liposomes was examined in U87 cells. These cells were exposed to either hypoxia-responsive or non-hypoxia-responsive liposomes under hypoxic (2% oxygen concentration) or normoxic (21% oxygen concentration) conditions for 2 h. Cells were then collected and DXR fluorescence analyzed by flow cytometry. Hypoxia-responsive liposomes presented a higher DXR fluorescence under hypoxic conditions compared to that under normoxic conditions. On the other hand, for non-hypoxia-responsive liposomes, the DXR fluorescence intensity was nearly the same for both conditions, demonstrating that presence of MDH is necessary for liposome destabilization under hypoxia.

To evaluate the biodistribution and the antitumoral efficacy of the formulation, a xenograft glioma model was obtained by intracranial injection of human glioblastoma U87 cells in Balb/c nude mice. The hypoxia-responsive liposomes encapsulating DXR and free DXR were injected intravenously and after 4 h animals were examined by an *in vivo* fluorescence microscopy imaging system. For animals treated with free DXR almost no DXR fluorescence was observed in the glioma, while a strong DXR fluorescence in the glioma of the animals treated with the hypoxia-responsive liposomes was observed. That demonstrated that the formulation could penetrate the blood-brain barrier delivering DXR to the tumor. The bioluminescence imaging was used to measure tumor growth. At day 30, the tumor growth rates in mice treated with non-hypoxia-responsive and hypoxia responsive liposomes were 2.72 fold and 0.79 fold, respectively, compared to day 10, and the median survival time were 55.5 and 65.5 days, respectively, demonstrating that the hypoxia-responsive liposomes exhibited higher antitumor efficacy in xenograft glioma model (135).

In another study, Liu et al. (136) used hypoxia responsive and non-hypoxia responsive liposomes as carriers of PLK1 siRNA for the treatment of glioma. The hypoxia responsive liposomes were composed of DSPE-PEG2000: CHOL: MDH (7,5:35:57,5 molar ratio) and non-hypoxia-responsive liposomes were composed of DSPE-PEG2000: CHOL: PD (7,5:35:57,5 molar ratio). The cellular uptake and intracellular distribution of these formulations were evaluated in hypoxic and normoxic conditions by fluorescence microscopy and flow cytometry in rat glial tumor C6 cells. The non-hypoxia-responsive liposomes showed slight and similar intracellular fluorescence intensity in C6 cells under hypoxic and normoxic conditions. Differently, the hypoxia-responsive liposomes showed higher intracellular fluorescence intensity compared to the non-hypoxia-responsive liposomes, especially under hypoxic conditions. The apoptosis-inducing effect was detected using the Annexin-V-FITC/PI in C6 cells. The hypoxia-responsive liposomes carrying siRNA showed 16.9% and 9% apoptotic ratio under hypoxic and normoxic conditions, respectively. The non-hypoxia-responsive liposomes carrying siRNA showed 5.5% apoptotic ratio under both oxygen conditions. The proliferation inhibition was evaluated by MTT assay. Hypoxia-responsive liposomes carrying siRNA inhibited the cell proliferation in 72.2% and 38.4% under hypoxic and normoxic conditions, respectively. The non-hypoxia-responsive liposomes carrying siRNA had only slight cell proliferation inhibition under both oxygen conditions. To evaluate the antitumoral efficacy of these formulations by bioluminescence imaging, a xenograft glioma model was obtained by intracranial injection of C6 cells to mice. Twenty-seven days after the different treatments, the tumor burden on animals treated with non-hypoxia-responsive liposomes was equal to 64.6% of that of the control (PBS) group. For animals treated with hypoxia responsive liposomes, tumor burden was 41.1% of the control group, suggesting this formulation is significantly superior on inhibiting the growth of glioma (136).

### ATP-Triggered Liposomes

In recent years, studies exploit the potential of ATP as a possible endogenous liposome trigger. This molecule is of extreme importance in cell signaling and metabolism and its concentration differs in extra and intracellular environments, allowing for the development of ATP-triggered drug delivery systems. In the extracellular fluid the ATP concentration is lower than 5 μM and double that amount is found inside the cells (14). Additionally, intratumoral interstitial ATP levels are between 10^3^-10^4^ fold higher when compared to levels found in normal tissues (137).

For the nanosystem to be sensitive to this ATP gradient, different strategies are possible. For example, the incorporation of single chain DNA aptamers, which are specifically recognized and activated by ATP. Another possibility is to use enzymes that use ATP as an energy source. The nanocarriers are than able to selectively release their drug content through a conformational switch under an ATP rich environment (138, 139). Although these concepts are already solid and elucidated, systems that use ATP as a stimulus for drug delivery are still under test of concept and need further studies to become a clinical reality. These studies include the investigation of ATP concentrations in the different organelles, the regulation of the glucose-dependent ATP response, and the discovery of aptamers capable of differentiating ATP from the adenosine diphosphate (ADP) molecule. In addition, it is necessary to evaluate the immunogenicity potentials of the bio-macromolecules used as ATP probes, since aptamers and enzymes are always made of DNA and proteins. However, like other endogenous triggers, the release of drugs into the ATP-stimulated tumor region has the advantage of not requiring any special equipment (139).

In order to exploit antitumor release using ATP-triggered liposomes, Mo et al. (140), have developed a fusogenic liposome composed of EPC : DOPE : CHOL (2.5:2.5:1 weight ratio) encapsulating DXR and a ATP-responsive aptamer (DXR-FL). This aptamer with high binding affinity to ADP/ATP was selected *in vitro* from a large set of random ssDNA sequences. A second liposome composed of EPC : DOTAP : CHOL (5:1:0.1 weight ratio) encapsulating ATP (ATP-L) was obtained and co-administered. The idea consisted on the co-internalization of these formulations in endosomes, followed by their pH-responsive membrane fusion and ATP-triggered DXR release and accumulation in the cell nucleus. When evaluated for its cytotoxicity against the human breast cancer cell line MCF-7, the IC50 of DXR-FL alone was 2.6 µg/ml while the IC50 of DXR-FL co-incubated with ATP-L was 1.5 µg/ml. When evaluated *in vivo* in a mouse model with MCF-7 tumor, DXR-FL co-delivered with ATP-L led to a higher tumor growth suppression compared to DXR-FL alone (tumor volumes around 0.25 cm³ and 0.35 cm³, respectively), confirming the effective role of extrinsic ATP in DXR release (140). In this study, extrinsic ATP is provided therefore it is a chemical exogenous trigger stimulus. Unfortunately, authors did not compare the DXR-FL to a non-ATP-sensitive formulation in order to compare to the potential of the endogenous ATP alone.

On **Table 2**, we summarize the most important aspects of important liposomal formulations triggered by different endogenous stimuli developed to date.

**Table 2:** Summary of important liposomal formulations triggered by different endogenous stimuli.

Trigger stimulus	Lipid composition	Encapsulated agent(s)	Encapsulated percentage	Mean diameter	Reference
**pH**	DOPE : HSPC : CHOL : CHEMS : ES-PEG-DSPE (at various molar ratio)	DXR	90%	151 nm	(106)
**pH**	DOPE : CHEMS : DSPE-PEG2000 (5,7:3,8:0,5) 40mM	DXR	25.5%	114.8 nm	(107)
**pH**	DOPE : CHEMS : DSPE-PEG2000: DSPEPEG2000-Fol (5,7:3,8:0,45:0,05) 20mM	DXR	97.6%	129 nm	(108)
**pH**	DPPC:mPEG-P(HPMA-g-His)-CHOL: biotin2-PEG (34:1:12,5 molar ratio)	DXR	84.7%	90 nm	(110)
**pH**	DOPE : CHEMS : DSPE-PEG : DSPE-PEG-H_7_K(R_2_)_2_. (65:30:4:1 molar ratio)	DXR	91%	92 nm	(111)
**GSH**	CHOL, SPC, DSPE-S-S-PEG5000 and DSPE-PEG2000-TAT (10:69.5:10:0.5 molar ratio)	PTX	84.30 ± 3.51%	102.70 ± 4.42 nm	(118)
**GSH**	SPC, CHOL, DOTAP, DOPE and CHOL-SS-mPEG at a 4:1:2:2:1 molar ratio, and hyaluronic acid 45mg	DXR	91.3 ± 3.2%	165.3 ± 0.2 nm	(122)
**GSH**	LHSSG2C14:SPC : CHOL 5:5:1	PTX) and anti-survinin siRNA	95.8 ± 2.8%	92.3 ± 1.6 nm	(120)
**Enzyme (**MMP-9**)**	LP1:DSPC (10:90 molar ratio)	5-carboxy fluorescein	N.A.	N.A.	(128)
**Enzyme** **(**MMP-9**)**	POPC: lipopeptide LP1: POPE-S-S-PEG5000: CHEMS: lissamine rhodamine lipid (59:30:5:5:1 molar ratio).	Gemcitabine	50%	86 nm	(129)
**Enzyme** **(**PLA2**)**	DSPC : DSPE-PEG : CHOL : DSPG (8:1:5:1 molar ratio) and DSPC : DSPE-PEG : CHOL : DSPE (8:1:5:1 molar ratio)	DXR	N.A.	100 nm	(131)
**Hypoxia**	DSPE-PEG2000: MDH: CHOL (MLP-DXR) (1:6:3 molar ratio).	DXR	8.7%	169.4 nm	(135)
**Hypoxia**	DSPE-PEG2000: CHOL: MDH (MLP-siRNA) (7,5:35:57,5 molar ratio)	SiRNA	N.A.	114 nm	(136)
**ATP**	DOPE : EPC:CHOL + ssDNA (2.5:2.5:1 weight ratio) DOTAP : EPC:CHOL (5:1:0.1 weight ratio)	DXR ATP	N.A 16.7%	195 nm 100 nm	(140)

## Concluding Remarks

The strategy of liposomal triggered release at the tumor site is part of liposomal evolution together with other strategies (e.g., long-circulating liposomes or active targeting) that aim on obtaining efficacious levels of drug in the tumor. Both the outer and inner tumor environment are being explored as means for liposomal triggered release. Herein, key studies concerning this topic were reviewed. As a relatively new strategy, its applications have not yet been fully developed, with a thermo-sensitive liposome being the only to make it to clinical studies so far. A special focus should be on liposomal formulations that are simple in composition, and that demand inexpensive trigger devices, thence enhancing the chances of clinical translation.

## Author Contributions

Conception or design of the work and figures: MF. Drafting the paper: MF, EG, MR, and MO. Revising it critically for important intellectual content: MF and MO. All authors contributed to the article and approved the submitted version.

## Funding

The authors would like to thank Conselho Nacional de Desenvolvimento Científico e Tecnológico – CNPq for supporting Mônica Cristina Oliveira with research grant (307098/2018-4).

## Conflict of Interest

The authors declare that the research was conducted in the absence of any commercial or financial relationships that could be construed as a potential conflict of interest.
